# Salt-Induced Diffusion of Star and Linear Polyelectrolytes within Multilayer Films

**DOI:** 10.1021/acs.macromol.3c00777

**Published:** 2023-07-10

**Authors:** Aliaksei Aliakseyeu, Parin Purvin Shah, John F. Ankner, Svetlana A. Sukhishvili

**Affiliations:** † Department of Materials Science & Engineering, Texas A&M University, College Station, Texas 77843, United States; ‡ Spallation Neutron Source Second Target Station Project, Oak Ridge National Laboratory, Oak Ridge, Tennessee 37831, United States

## Abstract

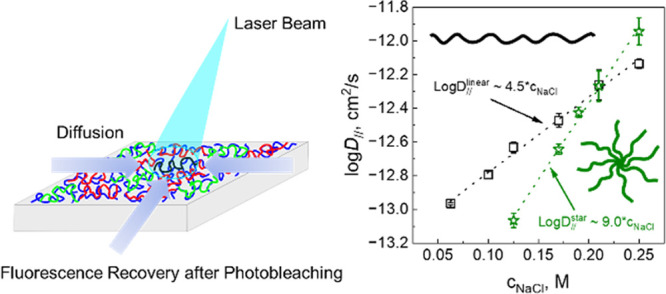

This study explores the effect of salt on the diffusivity of polyelectrolytes of varied molecular architecture in layer-by-layer (LbL) films in directions parallel and perpendicular to the substrate using fluorescence recovery after photobleaching (FRAP) and neutron reflectivity (NR) techniques, respectively. A family of linear, 4-arm, 6-arm, and 8-arm poly(methacrylic acids) (*L*PMAA, 4PMAA, 6PMAA, and 8PMAA, respectively) of matched molecular weights were synthesized using atom transfer radical polymerization and assembled with a linear polycation, poly[2-(trimethylammonium)ethyl methacrylate chloride] (QPC). NR studies involving deuterated QPC revealed ∼10-fold higher polycation mobility for the 8PMAA/QPC system compared to all-linear LbL films upon exposure to 0.25 M NaCl solutions at pH 6. FRAP experiments showed, however, that lateral diffusion of star PMAAs was lower than *L*PMAA at NaCl concentrations below ∼0.22 M NaCl, with a crossover to higher mobility of star polymers in more concentrated salt solutions. The stronger response of diffusion of star PMAA to salt is discussed in the context of several theories previously suggested for diffusivity of polyelectrolyte chains in multilayer films and coacervates.

## Introduction

Layer-by-layer (LbL) assemblies play an important role in engineering materials for drug delivery,^1,2^ photonics,^3^ microfluidics,^4,5^ and energy storage.^6−8^ It is thus critical to understand the response of these assemblies to different environments such as pH, salt concentration, and temperature. At a fundamental level, the response of LbL assemblies to environmental stimuli occurs via rearrangements of polymer conformations in the films and/or changes in the number of intermolecular contacts with partner molecules, both impacting the spectrum of molecular motions. Up to now, the diffusional response of assembled polyelectrolytes has mostly been studied for the case of linear polymers.

The mobility of polyelectrolytes assembled within LbL films is primarily determined by their chemical nature and the strength of intermolecular ionic pairing.^9−11^ For example, Soltwedel et al. showed that diffusion of poly(styrene sulfonate) (PSS) was faster in assemblies with poly(diallyldimethylammonium chloride) (PDADMAC) than with poly(allylamine hydrochloride),^9^ due to the difference in the intrinsic strength of ionic pairing^12^ and the linear charge density mismatch in these systems. Further, Xu et al. showed that the steric bulk of quaternary polyamines resulted in faster interdiffusion of PSS in polyelectrolyte multilayers (PEMs) due to weaker binding between polyanion and the sterically hindered polycation.^10^ Another important parameter that affects chain mobility in LbL films is molecular weight.^13−16^ Typically, polyelectrolytes with low molecular weights demonstrate non-linear film growth compared to their higher molecular weight counterparts due to increased molecular diffusivity.^14,17^ For weakly interdiffused, linearly depositing films of poly(methacrylic acid) (PMAA) and a polycationic partner, Xu et al. found that the lateral center-of-mass diffusion coefficient (*D*) scaled with the molecular weight of the co-assembled PMAA as *D* ∼ *M*_w_^–1^ even for long polyacid chains, suggesting that chain mobility in LbL assemblies does not necessarily follow the generally accepted laws of bulk diffusion of entangled polymers.^13^

Note that the molecular response of LbL-assembled polyelectrolytes is largely enabled by the fact that salt can strongly enhance molecular mobility via an increase in the fraction of extrinsically compensated polyelectrolyte charge.^18−20^ Earlier, atomic force microscopy (AFM) studies showed that the annealing of LbL films in salt solutions led to smoother surface morphologies due to enhanced mobility of charge-paired polyelectrolyte chains.^19,21^ Later studies quantitatively explored salt-enhanced polymer mobility perpendicular to the surface using neutron reflectivity (NR)^16,18,22^ and parallel to the surface using fluorescence recovery after photobleaching (FRAP). Moreover, studies that use a combination of NR and FRAP techniques have also been reported.^23−25^ The most general observation in these studies was an enhancement of polymer mobility by salt ions and a higher mobility and stronger response to salt of polymer motions parallel to the substrate. Additionally, salt was demonstrated to enhance diffusion of weak poly(carboxylic acid)s in both spin-coated and dip-coated films, but polymer diffusion in spin-coated films was restricted due to increased ionic pairing and chain flattening induced by the centrifugal forces during deposition.^23^

In contrast to linear polyelectrolytes, the diffusion of non-linear polymers in PEMs remains largely unexplored. One important class of non-linear polymers, that is, star polymers, features a single branching point (core) with attached linear chains (arms).^26^ This unique molecular architecture leads to distinct properties such as compact molecular size and lower intrinsic viscosity as compared to linear chains and enhanced segmental density within the core.^26^ Star polymers are used in novel lubricant additives,^27,28^ thin films for energy dissipation during ballistic impact,^29,30^ and as nanocontainers for drug^31−33^ and gene delivery^34^ due to enhanced small-molecule trapping efficiency. The latter property is crucial for engineering novel LbL coatings with an increased payload of drug molecules.^1,31,35^ Yet, little is known about the response of star polyelectrolyte assemblies to environmental stimuli. Earlier studies of star-containing LbL films focused on the effect of polymer architecture on the formation of PEMs.^36−40^ Additionally, several studies reported changes in the external roughness of star-containing LbL films by post-assembly treatment by solutions at different pHs.^36,37^

Recently, we explored the effect of polymer architecture on the diffusion of polymer chains using NR and *in situ* ellipsometry techniques. The systems of interest included assemblies of star poly(acrylic acid)s with a linear polycation at low pH, where ionization of the weak polyelectrolyte stars was largely suppressed, contributing to differences in mobility of star and linear polyacids perpendicular to the substrate.^41^ In this paper, we employ NR and FRAP to investigate the diffusion of polymers perpendicular and *lateral* to the surface, respectively. Faster diffusion of polymer chains parallel to the surface and the neutral pH values chosen for these experiments enabled observation of the mobility of assembled polyelectrolytes of varying molecular architectures over a wide range of salt concentrations, allowing us to establish quantitative relationships between the molecular architecture of weak polyelectrolytes, their diffusivity within LbL films, and the alteration of this diffusivity upon exposure to salt ions.

## Materials and Methods

### Materials

Pentaerythritol (synthesis grade) and dipentaerythritol (synthesis grade) used for synthesis of tetra and hexa-functional initiators were received from Merck. Tripentaerythritol (technical grade) used for synthesis of an octa-functional initiator was purchased from Sigma-Aldrich. Branched poly(ethylene imine) (BPEI), ethyl α-bromoisobutyrate (EtBiB) (98%), anisole, α-bromoisobutyril bromide (98%), tert-butyl methacrylate, sodium hydroxide, isopropanol (ACS grade), dimethylformamide (DMF, ACS grade), copper(II) bromide (99%), tris(2-pyridylmethyl)amine (TPMA), tin(ii) 2-ethylhexanoate (Sn(Oct)_2_, 92.5–100%), deuterated chloroform (CDCl_3_), and *d*_6_-DMSO were purchased from Sigma-Aldrich. Aluminum oxide acidic (50–200 μm, 60 Å) and aluminum oxide basic (40–300 μm, 60 Å) of chromatographic grade were obtained from Acros Organics. Chloroform (ACS grade), ethanol (ACS grade), hydrochloric acid (36.5%, ACS grade), sodium bicarbonate (ACS grade), and dichloromethane (ACS grade) were purchased from VWR. Trifluoroacetic acid (TFA) (99%) and pyridine (99%) purchased from Alfa Aesar. All chemicals were used as received. Dialysis tubing (cutoff 3.5 kDa) and Alexa Fluor 488 hydrazide were purchased from Thermo Fisher Scientific. Water used in this study was purified using a Millipore Milli-Q system. Hydrogenated poly[2-(trimethylammonium)ethyl methacrylate chloride] (*h*QPC, *M*_n_ 30 kDa, *Đ* < 1.2) and deuterated QPC (*d*QPC, *M*_n_ 35 kDa, *Đ* < 1.2) were synthesized and characterized as described in our previous publications.^11,24^ Synthesis and characterization of tetrakis(2-bromoisobutyrate) (4f-BiB), dipentaerythritol hexakis(2-bromoisobutyrate) (6f-BiB), and tripentaerythritol octakis(2-bromoisobutyrate) (8f-BiB) were described in our previous publications.^41,42^

### Synthesis of Linear and Star Poly(*tert*-butyl methacrylates)

Linear and star poly(*tert*-butyl methacrylates) (PTBMA) were synthesized using activators regenerated by electron transfer atom transfer radical polymerization (ARGET ATRP), as described previously for methacrylate monomers.^42,43^ The proportions of initiators, copper(II) bromide, Sn(Oct)_2_, TPMA, and TBMA are shown in Table 1. The concentration of the monomer was kept constant (3 M solution of TBMA in anisole). The temperature of polymerization was maintained at 40 °C. Polymerization kinetics was monitored by taking aliquots of the solution mixture, followed by gel permeation chromatography (GPC, Tosoh EcoSec) analysis using DMF as an eluent (Figures S1–S4).

**Table 1:** Molar Ratios of Initiating Sites, Copper(II) Bromide, the Ligand, the Reducing Agent, and the Monomer to the Initiator in the Polymerization Mixtures

	initiator	initiating sites	CuBr_2_	TPMA	Sn(Oct)_2_	monomer
EtBiB	1	1	0.12	0.6	1.2	1000
4f-BiB	1	4	0.07	0.35	0.7	1000
6f-BiB	1	6	0.07	0.35	0.7	1000
8f-BiB	1	8	0.07	0.35	0.7	1000

**Figure 1:**
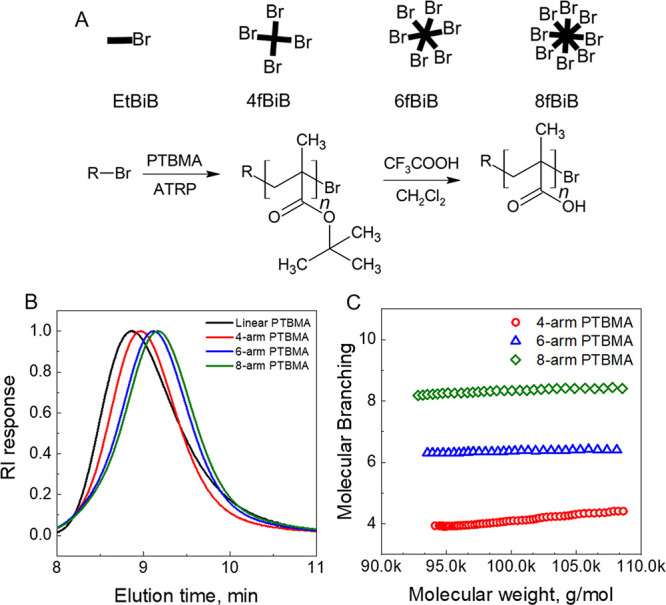
(A) Schematics of polymerization of linear and star PTBMAs and deprotection to obtain linear and star PMAAs. *R* represents initiators used in polymerization, that is, EtBiB for linear PTBMA, 4f-BiB for 4-arm, 6f-BiB for 6-arm, and 8f-BiB for 8-arm star PTBMAs. (B) GPC traces of linear, 4-, 6-, and 8-arm PTBMA in DMF (flow rate 0.2 mL/min) as monitored by an RI detector. (C) Branching per molecule for 4-, 6, and 8-arm PTBMAs determined using Wyatt Viscostar III and Wyatt miniDAWN detectors.

All polymers were then dissolved in DMF and characterized using GPC equipped with multiangle laser light scattering and viscometry detectors, which were precalibrated using a 30 kDa polystyrene standard. The specific refractive index increments, *dn/dc*, were determined for all polymers using a refractive index (RI) detector as described elsewhere.^42^ The determined number-average (*M*_n_), weight-average (*M*_w_) molecular weights, dispersity (*Đ*), and degrees of branching are shown in Table 2, while the GPC traces and determined branching degrees for the linear and star polymers are presented in Figure 1.

**Table 2:** Molecular Characteristics: Weight-Average Molecular Weight (*M*_w_), Dispersity (*Đ*), Polymer Branching, and Number of Monomer Units of Linear and Star PTBMAs as Determined by GPC in Conjunction with Light Scattering and Viscometry Analysis

polymer	*M*_w_, kDa	*Đ*	branching	monomer units
*L*PTBMA	98.7	<1.1		695
4PTBMA	107.6	<1.1	4.1 ± 0.2	758
6PTBMA	110.1	1.14	6.1 ± 0.1	776
8PTBMA	109.0	1.15	8.1 ± 0.1	767

### Synthesis of Linear and Star Poly(methacrylic acids)

The deprotection procedure of linear and star PTBMAs was adapted from previous publications.^33,41^ In brief, 0.6 g (0.004 mol of polymer units) of linear or star PTBMA was dissolved in 15 mL of dichloromethane in a 20-mL glass vial under vigorous stirring. Then 1 mL (0.013 mol) of TFA was added to the vial under continuous stirring and stirred overnight. The precipitated polymers were filtered and dissolved in water. The aqueous solutions were placed in dialysis tubes (cut off *M*_w_ = 3.5 kDa) and dialyzed against 0.001 M aqueous HCl, which was replaced every 12 h for a total of 2 days. The solutions were then freeze-dried to yield powders of linear and star PMAAs, whose ^1^H NMR analysis in *d*_6_-DMSO confirmed complete disappearance of the tert-butyl groups (Figure S5).

### Multilayer Deposition and Salt Stability Studies

Multilayers of linear and star PMAAs and QPC were deposited on silicon substrates using the LbL dip deposition technique. The silicon wafers were cleaned as described elsewhere^25^ and primed by adsorption of a BPEI monolayer by immersion in 0.2 mg/mL BPEI solution at pH 6 for 15 min. LbL films were then constructed via alternating adsorption of 0.2 mg/mL linear or star PMAA and QPC from solution in 0.01 M phosphate buffer at pH 6 and rinsed for 30 s in 0.01 M phosphate buffer at pH 6 between deposition of the polycation and polyacids.

10-bilayer QPC/PMAA LbL films were used in the salt stability experiments. The stability studies were performed by measuring the thicknesses of the dry films prior to and after exposure to salt solutions. After each exposure, the films were rinsed in 0.01 M phosphate buffer solution (pH 6) and dried with dry nitrogen gas before measuring their thickness. The measurements were conducted for a salt concentration range of 0–2.7 M NaCl with 0.25 M NaCl and 0.1 M NaCl concentration increments in the 0–2 M and 2–2.7 M NaCl ranges, respectively.

The kinetic stability studies were performed by exposing the LbL films to 2.4–2.6 M NaCl solutions and monitoring the time evolution of film dissolution by periodically measuring film thickness by ellipsometry.

### Ellipsometric Measurements

The thicknesses of the deposited films in the dry state were characterized using a variable-angle spectroscopic ellipsometer (M-2000 UV–visible-NIR (370–1000 nm) manufactured by J. A. Woollam Co., Inc., Lincoln, NE, USA) at four angles of incidence: 45°, 55°, 65°, and 75°. Swelling of PMAA/QPC films was measured in situ using a liquid cell equipped with a temperature controller on the same ellipsometer. All data were fitted using the Cauchy model, as described in our previous publication.^44^

### Neutron Reflectometry

Neutron reflectometry studies were performed on the Spallation Neutron Source Liquids Reflectometer at Oak Ridge National Laboratory using experimental parameters described earlier.^24,41,44^ LbL films for NR studies were assembled on BPEI-primed Si wafers in the following manner: BPEI/PMAA/(*h*QPC/*L*PMAA)_4_/(*d*QPC/*L*PMAA)_3_ for the linear PMAA/QPC system and BPEI/PMAA/(*h*QPC/xPMAA)_3_/(*d*QPC/xPMAA)_3_ for the star PMAA/QPC systems, where x indicates the number of arms of the star polymer (4, 6, and 8 for 4PMAA, 6PMAA, and 8PMAA, respectively). The neutron reflectometry data were fitted using a model that was previously described elsewhere.^41,45,46^ The specific parameters used for fitting of NR data are given in the Supporting Information.

### Fourier Transform Infrared Spectroscopy

30-bilayer QPC/PMAA LbL films were deposited onto undoped silicon wafers, as described in the Multilayer Deposition and Salt Stability Studies section, and used in transmission FTIR measurements. All samples were analyzed with a Tensor II spectrophotometer (Bruker Optics GmbH, Germany). The FTIR band in the 1500–1800 cm^–1^ region was deconvoluted into three Gaussian peaks centered at 1560 cm^–1^ (asymmetric >COO^–^ stretching vibrations), 1710 cm^–1^ (carbonyl vibration of non-ionized >COOH), and 1735 cm^–1^ (ester group of the polycation) using the Origin Lab 2017 program. To quantify ionization degree, absorbances of the bands corresponding to asymmetric >COO^–^ stretching vibrations at 1560 cm^–1^ were compared with those of the carbonyl vibration of the non-ionized >COOH group at 1710 cm^–1^.

### Labeling of Linear and Star PMAAs with Alexa 488

The labeling of linear and star PMAAs was performed following a previously developed procedure.^47^ In brief, 1 mg (1.2 × 10^–5^ moles) of linear or star PMAA was dissolved in 0.1 mL of 0.1 M sodium dihydrophosphate buffer at pH 5. Then, separately prepared 25 mg/mL solutions of 1-ethyl-3-dimethylaminopropyl-carbodiimide hydrochloride (1.3 × 10^–5^ moles, 0.1 mL) and N-hydroxysulfosuccinimide sodium salt (1.4 × 10^–5^ moles, 0.12 mL) in the same buffer were added to the solution of polyacid and left for an hour for activation. Then, 1 mg of Alexa 488 hydrazide was dissolved in 0.1 mL of 0.1 M sodium dihydrophosphate buffer at pH 5 and 0.025 mL (4.4 × 10^–4^ mol) of this solution was added to the PMAA solution after the activation step. The mixtures were left overnight in darkness. After that, the mixtures were diluted with 2 mL of 0.01 M phosphate buffer at pH 7, placed into a dialysis tube (molecular weight cutoff 3.5 kDa), and dialyzed sequentially against 0.001 M HCl (∼5 times) and 0.1 M NaCl (∼5 times) and then DI water (∼20 times) for at least 1 month in darkness. The dialysis continued until there was no fluorescence detected in the surrounding water as measured by fluorimetry (measured by Shimadzu RF-6000) and no fast-diffusing free Alexa 488 species as detected by fluorescence correlation spectroscopy (Figure S12) using the setup described in our previous work.^47^ The efficiency of labeling was calculated using UV–vis analysis (measured by a Shimadzu UV 2600 spectrophotometer) assuming that free Alexa 488 and Alexa attached to polymer chains have the same extinction coefficient of 71,000 cm^–1^ M^–1^. This analysis showed that the labeled linear PMAA (LPMAA*), as well as 4-, 6-, and 8-arm star PMAAs (4PMAA*, 6PMAA*, and 8PMAA*, respectively) contained 1 label per 790, 1060, 830, and 1030 units for LPMAA*, 4PMAA*, 6PMAA*, and 8PMAA*, respectively. All solutions were stored in water at 0.1 mg/mL concentration in the fridge.

### FRAP Experiments

LbL films were assembled in a home-made glass cell and measured using a custom FRAP setup described in our previous publications.^11,25^ Prior to deposition, a 0.17 mm glass cover (25 mm in diameter, EMS, US) and a 5-mm borosilicate glass Raschig ring (Sigma-Aldrich) were immersed in concentrated sulfuric acid for 20 min, thoroughly rinsed with DI water, and dried under nitrogen flow. After that, the Raschig ring was attached to the glass cover using thermo wax (Quick Stick 135 Mounting Wax, EMS) at elevated temperature. The constructed cell was left overnight at room temperature. The internal glass surface of the constructed cell was then primed by adding 0.2 mg/mL BPEI solution at pH 6 into the cell for 15 min, followed by rinsing with 0.01 M phosphate buffer at pH 6. Then 0.2 mg/mL solutions of PMAA, PMAA*s, and QPC in 0.01 phosphate buffer at pH 6 were used to prepare the LbL films. Each solution was left in the cell for 5 min, and the cell was filled and rinsed 3 times (30 s for each rinsing cycle) with 0.01 M phosphate buffer at pH 6. After the construction of BPEI/PMAA/(QPC/PMAA)_2_/(QPC/PMAA*)_3_/(QPC/PMAA)_2_ multilayers containing fluorescently labeled PMAA* in the middle, the films were dried in a nitrogen gas flow for 2 min prior to performing FRAP experiments.

All FRAP experiments were performed with films exposed to salt/buffer solutions using the custom-built setup described elsewhere.^13,24,25^ The radius of the bleaching spot (*R* = 0.28 μm) was determined using an Alexa 488 solution at pH 6 assuming that the label had a diffusion coefficient of 440 μm^2^/s, as determined previously.^48^ The experimental cells containing deposited multilayers were filled with 120 μL of 0.01 M phosphate buffer containing from 0.0625 to 0.25 M NaCl and were covered with a glass slide to prevent solution evaporation. A spot on the films was bleached for 3 s by a focused laser beam at 0.1 mW. The spot was bleached to 25–30% of its initial fluorescent intensity to avoid possible side reactions in the film, such as photo-induced crosslinking. After bleaching, recovery of the fluorescence intensity in the bleached area was recorded every 2–20 min at a much lower power of 1 μW to avoid additional photobleaching. Since fluorescence recovery time in all experiments was much longer than the bleaching time (e.g., > 0.5 h vs 3 s), the contribution of molecular motions occurring during sample bleaching to fluorescence intensity recovery profiles was negligible. All experiments were repeated at least twice.

## Results and Discussion

To explore the effect of the molecular architecture of polyacids on polymer mobility within LbL films, we first synthesized a family of star PTBMAs (Figure 1) via core-first ARGET ATRP polymerization that was previously applied to synthesize poly(methacrylate)-based block copolymers^43^ and temperature-responsive star polymers.^42^ ARGET ATRP provides good control over polymer molecular weight, dispersity (*Đ*),^49^ and branching^42^ and reduces the volume of organic solvent (in this case anisole) used in the synthesis in comparison to traditional ATRP.^50^

Figures S1–S4 show the kinetics of polymerization of tert-butyl methacrylate using linear and star initiators. For all systems, we observed a linear change of monomer concentration over time that is consistent with the controlled nature of the polymerization. We further determined absolute molecular weights and degrees of branching of the synthesized star polymers using a combination of multiangle light scattering and viscometry techniques, following the protocol described in our previous publications.^41,42^Figure 1A shows the elution times of linear and star PTBMAs, and Figure 1B shows polymer branching, confirming successful synthesis of well-defined star polymers. The molecular characteristics of linear and star PTBMA are summarized in Table 2. PTBMA polymers were then deprotected using TFA in dichloromethane (see the Materials and Methods section) to yield a family of linear (*L*PMAA) and star PMAA with 4, 6, and 8 arms (4PMAA, 6PMAA, and 8PMAA, respectively) of matched molecular weights. Proton NMR analysis confirmed the completeness of deprotection of the PTBMA polymers (Figure S5), yielding a series of polyacids with varying molecular architecture.

**Figure 2:**
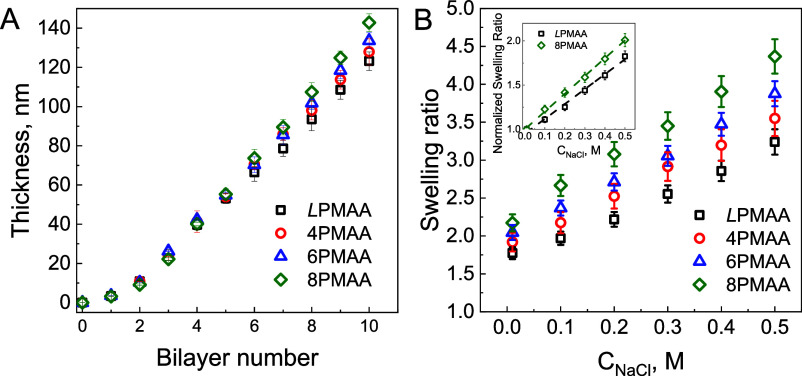
(A) Ellipsometric thicknesses of dry *L*PMAA/QPC (squares), 4PMAA/QPC (circles), 6PMAA/QPC (triangles), and 8PMAA/QPC (diamonds) films constructed from 0.2 mg/mL solutions in 0.01 M phosphate buffer at pH 6. (B) Swelling ratios of (*L*PMAA/QPC)_7_, (4PMAA/QPC)_7_, (6PMAA/QPC)_7_, and (8PMAA/QPC)_7_ films at different salt concentrations at pH 6. The inset shows swelling ratios at different salt concentrations normalized to film swelling in the absence salt for (LPMAA/QPC)_7_ and (8PMAA/QPC)_7_ films. The dashed line is

Figure 2A shows growth curves of the electrostatic assembly of linear and star PMAAs with a polycation (QPC) as determined by ellipsometry. All films grew nonlinearly up to four bilayers and then switched to a linear regime, similar to the previously reported deposition of films of linear PMAA and QPC.^44^ Note that all the films with star PMAA were thicker than their linear *L*PMAA/QPC counterpart, with a maximum of ∼15% film thickness difference for the most branched 8PMAA polymer. Previous studies of PMAA/QPC films assembled with linear chains have shown that film thickness and degree of intermixing can be controlled by varying pH^11^ and time of deposition.^44^ Faster growth of star-containing films may be due to a larger quantityof star polymers being adsorbed to the film surface,^51^ or to enhanced polymer mobility of star polymers having a lower number of contacts with linear polymers.^39,41^ Here, due to the short (5 min) deposition times and low-salt conditions, the faster growth of star-containing films was likely due to the larger mass of star polymers adsorbed at the film surface.

We next aimed to explore the swelling properties of the deposited films and their response to salt in an aqueous environment. Figure 2B shows swelling ratios for *L*PMAA/QPC and star PMAA/QPC films in the absence and in the presence of NaCl. The star-containing LbL films swelled more than *L*PMAA/QPC films at all salt concentrations and were more sensitive to salt concentration (e.g., ∼10% greater increase in swelling at 0.5 M NaCl for 8PMAA/QPC films in comparison to *L*PMAA/QPC), suggesting that molecular architecture affected both the density of ionic pairing in low-salt solutions, as well as the ease of dissociation of the ionic pairs as salt concentration was increased. This result is consistent with a lower density of ionic contacts in star-containing films.

In addition to equilibrated swelling behavior, we were also interested in the kinetics of swelling at different salt concentrations. Tanchak and Barrett described the swelling kinetics of poly(acrylic acid)/(poly allylamine hydrochloride) multilayers in terms of the solvent uptake and the relaxation of polymer chains.^52^ The swelling kinetics of LbL films can be analyzed using the diffusion equation , where *M_t_* represents an increase of the film thickness at time *t*, *M*_∞_ is film thickness in the equilibrium state, *k*_1_ is a characteristic constant, *t* is time, and *n* is the exponent that describes the diffusion type (*n* = 0.5 for Fickian diffusion when the rate of permeant diffusion is much less than the polymer segment mobility, *n* > 1.0 for non-Fickian diffusion when the rate of permeant diffusion exceeds that of the polymer segment mobility, and 0.5 < *n* < 1.0 for anomalous diffusion). Analysis of the swelling kinetics in our system shown in Figure S6 and Table S1 yielded n values between 1.1 and 1.6 for LbL films of linear and star PMAAs at different salt concentrations, suggesting that film swelling was controlled by diffusion of solvent and counterions rather than relaxation of polymer chains.

**Figure 3:**
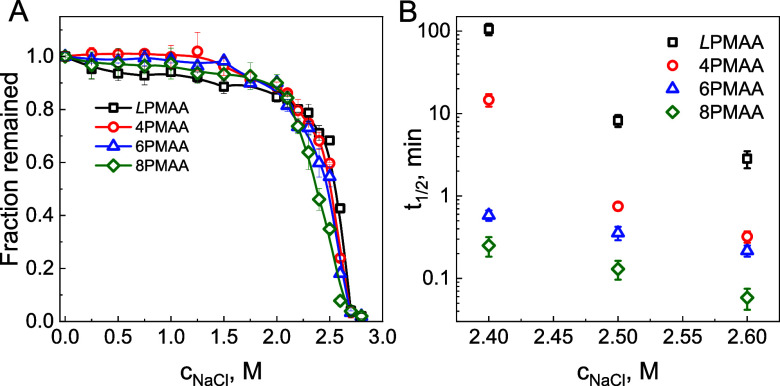
(A) Salt stability of (*L*PMAA/QPC)_10_ (squares), (4PMAA/QPC)_10_ (circles), (6PMAA/QPC)_10_ (triangles), and (8PMAA/QPC)_10_ (diamonds) films presented as normalized ellipsometric dry thicknesses retained at the surface upon 5-min exposures of the films to increasing concentration of NaCl at pH 6. The solid lines are only for eye guidance. (B) Characteristic film dissolution half-time as a function of salt concentration for (*L*PMAA/QPC)_10_, (4PMAA/QPC)_10_, (6PMAA/QPC)_10_, and (8PMAA/QPC)_10_ films. The kinetic curves of film decomposition are shown in Figure S7.

We further explored the stability of the assembled films at elevated salt concentrations. Figure 3A shows that all films were stable up to 2 M NaCl but started to decompose at higher salt concentrations. Note that 8PMAA/QPC films showed the lowest resistance to salt and decomposed at 2.4 M NaCl, while a decrease in the number of arms led to increased film stability in higher salt conditions. Studies of the kinetics of film decomposition in 2.4–2.6 M NaCl solutions (Figure 3B) showed that for all the films, salt disrupted ionic pairing and decreased the half-life of film decomposition (calculated as the time when 50% of the film thickness remained on the substrate), with faster decomposition of the star-containing LbL films consistent with a greater swelling of these films upon exposure to salt.

**Figure 4:**
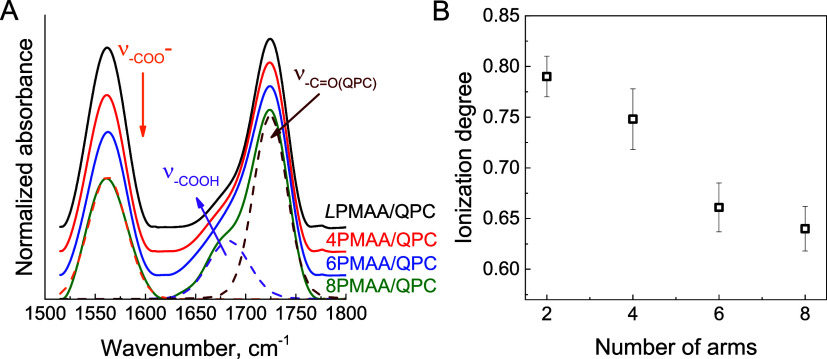
(A) FTIR transmission spectra of (from top to bottom) (*L*PMAA/QPC)_30_, (4PMAA/QPC)_30_, (6PMAA/QPC)_30_, and (8PMAA/QPC)_30_ films deposited on Si wafers at pH 6. The dotted lines represent peak deconvolutions for 8PMAA/QPC PEMs. (B) Degrees of ionization of linear and star PMAA calculated as (*A*_νCOO^–^_/[*A*_νCOO^–^_ + *A*_νCOOH_]) for 30-bilayer PMAA/QPC films deposited at pH 6, where *A*s are the absorbances of the corresponding bands.

Differences in binding between linear and star PMAA with QPC were also revealed through FTIR studies of the ionization of assembled PMAAs. The stretching vibrations of non-ionized and ionized carboxylic groups of poly(carboxylic acids) occur at different wavenumbers ∼1690 and 1560 cm^–1^, respectively,^41^ and can be used to determine the ionization of the assembled polyacids assuming equal extinction coefficients of the respective bands.^41,53^Figure 4A shows FTIR spectra for *L*PMAA/QPC, 4PMAA/QPC, 6PMAA/QPC, and 8PMAA/QPC films. While visual inspection of the data shows that the relative absorbance of the peak at 1560 cm^–1^ decreased for the films of more highlybranched PMAAs, spectral deconvolution in the 1500–1770 cm^–1^ region using a three-peak model (the ionized and non-ionized carboxylic groups at 1560 and 1690 cm^–1^ and the ester group in QPC at 1725 cm^–1^) allowed us to quantify the ionization of the assembled polyacids. Figure 4B shows that ionization of PMAA was ∼20% higher in *L*PMAA films relative to 8PMAA films, indicating that star PMAAs formed fewer ionic pairs with QPC due to their branched architecture, in agreement with the data in Figures 2 and 3. It is plausible that polymer segments within the cores of the sPMAA remain unbound with the polycation, and that the fraction of units unreachable by QPC increases with increasing degree of polyacid branching.

Different expectations can be formulated of the effect of intermolecular contacts and molecular architecture on polymer chain diffusivity. For example, Tsukruk and co-workers suggested that the lateral diffusion of star polymers in LbL films can be hindered due to fast complexation of star polymers during film deposition.^39^ Here, we directly measured the lateral diffusion (*D*_*∥*_*)* of fluorescently labeled star PMAAs using FRAP to explore the dependence of molecular mobility on polymer architecture. Conversely, diffusion of the polycationic partners perpendicular to the substrate (*D*_*⊥*_) was followed using NR.

**Figure 5:**
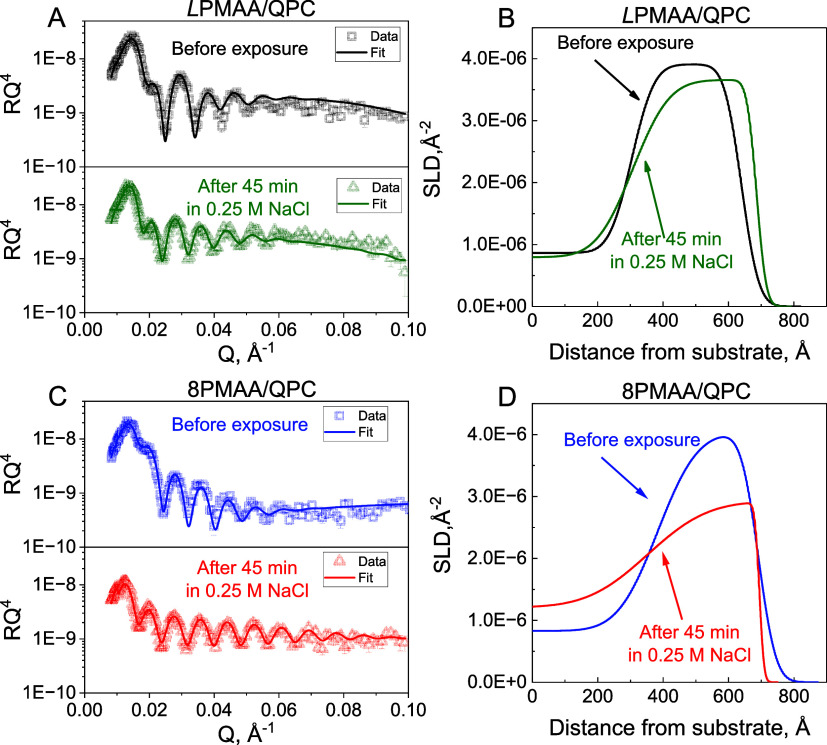
Neutron reflectivity data (plotted as RQ^4^ to enhance small features) and the corresponding neutron scattering length density profiles for *L*PMAA/(*h*QPC/*L*PMAA)_4_(*d*QPC/*L*PMAA)_3_ (A,B) and 8PMAA/(*h*QPC/8PMAA)_3_(*d*QPC/8PMAA)_3_ (C,D) LbL films deposited at pH 6 before and after 45-min exposure to 0.25 M NaCl solutions. The NR data and the fits for other time scales and systems are shown in Figures S8–S11.

Figures 5 and S8–S11 show the results of NR measurements of PMAA/QPC films constructed using hydrogenated QPC deposited within the lower layers and deuterated QPC (*d*QPC) within the upper film layers (see Materials and Methods and Figure 5 caption), and the data were fitted using a model described in our recent publication.^41^ The model takes into account the fractions of water (*w*_H_2_O_), polycation (*f*_QPC_), and polyanion (*f*_PMAA_) within the film and the fraction of deuterated polymer (*w*_dQPC_) within the hydrogenated and deuterated layer stacks (H- and D-stacks, respectively) and the density of the film (*ρ*) (Tables S3–S20). The fitted scattering length density profiles show that as-deposited films retained different degrees of stratification (Figures 5 and S8–S11), and that no *d*QPC was found in the H-stacks (Tables S3, S8, S13, and S17). However, the internal roughness (σ_int_) almost doubled from (12.8 ± 1 nm) for *L*PMAA/QPC to (22.6 ± 4 nm) for 8PMAA/QPC films (Tables S3 and S17).

**Figure 6:**
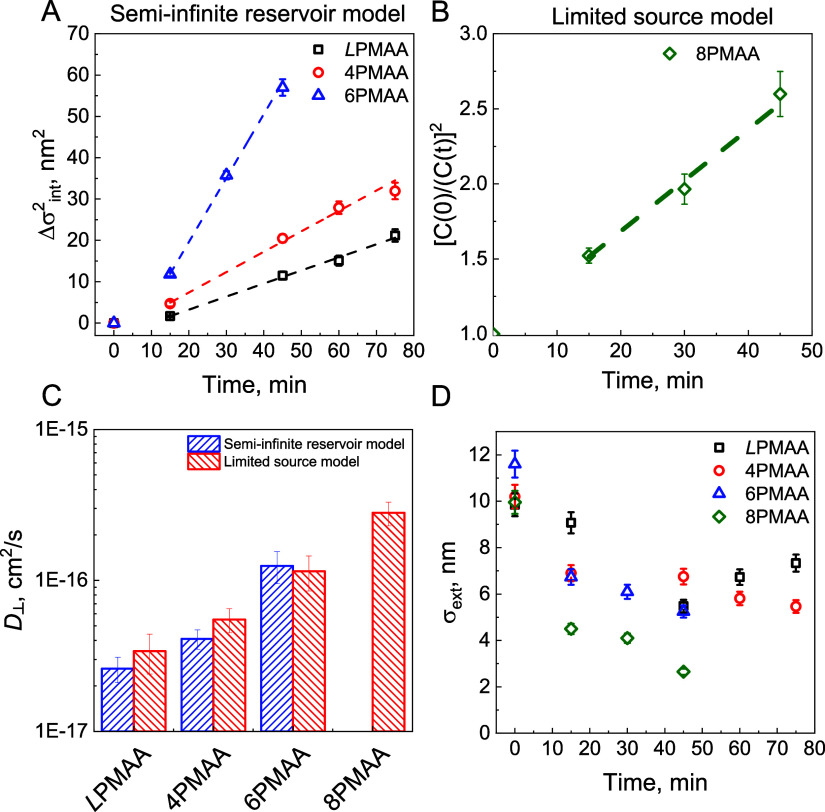
(A) Time evolution of the square of internal roughness (σ^2^_int_) for *L*PMAA/QPC (squares), 4PMAA/QPC (circles), and 6PMAA/QPC (triangles) systems upon exposure to 0.25 M NaCl solutions. (B) [C(0)/C(t)]^2^ vs time for 8PMAA/QPC films. (C) Diffusion coefficients of QPC in the linear- and star-containing polymer films estimated using the semi-infinite reservoir and limited-source models. (D) External roughness as a function of salt annealing time for *L*PMAA/QPC (squares), 4PMAA/QPC (circles), 6PMAA/QPC (triangles), and 8PMAA/QPC (diamonds) films.

Polymer interdiffusion within the films was then induced via annealing in 0.25 M NaCl at pH 6. The responses of the films containing linear and star PMAA to salt were drastically different (Figures 5 and 6A). For *L*PMAA/QPC films, the rate of change in the width of the interface between the H-stack and the D-stack was relatively slow (∼0.3 nm^2^/min), indicating slow interdiffusion of *d*QPC into the H-stack (Tables S3–S7), while in 6PMAA/QPC films, intermixing of the H- and D-stacks was ∼6-fold faster.

The diffusion coefficients of *d*QPC were then estimated using either the semi-infinite reservoir or limited-source models. The semi-infinite reservoir model assumes an unlimited quantity of diffusing material in the donor stack whose concentration remains unaffected by the progressing diffusion. This model cannot be used in cases where significant film intermixing occurrs.^54^ In such cases, the limited-source model accounts for an exchange of material between both stacks and relates the diffusion coefficients with changes in stack polymer concentrations. For *L*PMAA/QPC, 4PMAA/QPC, and 6PMAA/QPC, we did not observe significant exchange of deuterated and hydrogenated polymers between stacks (Table S21) and so used the semi-infinite reservoir model to determine *D*_*⊥*_ as σ_int_^2^/2*t*, where *t* is exposure time (Figure 6A). However, this model could not be used for the 8PMAA/QPC system due to strong intermixing between *h*QPC and *d*QPC at early times of salt annealing. In this case, we used the limited-source model,^10,18,41^ which correlates changes in concentration of *d*QPC in the stacks at different diffusion times as , where *C*(0) is the relative concentration of *d*QPC in the D-stack at zero time, *C*(*t*) is relative concentration of *d*QPC in a D-stack at a given time of exposure, and σ_0_ is the initial internal roughness between stacks. Figure 6B shows that scaled linearly with time for the 8PMAA/QPC system, yielding  as a slope. The diffusion coefficients of *d*QPC in *L*PMAA/QPC, 4PMAA/QPC, and 6PMAA/QPC were determined using the limited-source model, where we were able to confidently fit the *f*_QPC_ in the H-stacks. Figure 6C shows that the diffusion coefficients obtained from both models were similar, suggesting that these models can be used interchangeably for estimating diffusion coefficients in our system. The values of QPC diffusion coefficients are comparable with those reported for salt-induced diffusion of *d*QPC in a PMAA/QPC system of linear polyelectrolytes,^24^ because both calculations were done for non-swollen films. At the same time, the obtained values are at least one order magnitude lower than those for diffusion of QPC chains within QPC/PMAA films measured from solution.^44^ Compared to PSS/PDADMAC LbL films,^18,22^ the diffusion coefficients in this work are higher, because PSS is known to form stronger ionic pairs relative to poly(carboxylic acids).^18,22^ When comparing the values of diffusion coefficients in different systems, the results clearly show that equivalent molecular weight 8-arm branched PMAA features approximately an order of magnitude greater polycation mobility in the direction perpendicular to the substrate. Note that for all the films, exposure to high-salt conditions resulted in a decrease in external film roughness (Figure 6D), in agreement with previous observations (made by AFM) of smoothening of film surfaces in high-salt environments due to increased diffusivity of polymer chains.^19,21^ The decrease in surface roughness was more significant in the case of star-containing films, and specifically for 8PMAA stars, suggesting an increased diffusivity of star polymers in high-salt solutions relative to linear polyacids.

**Figure 7:**
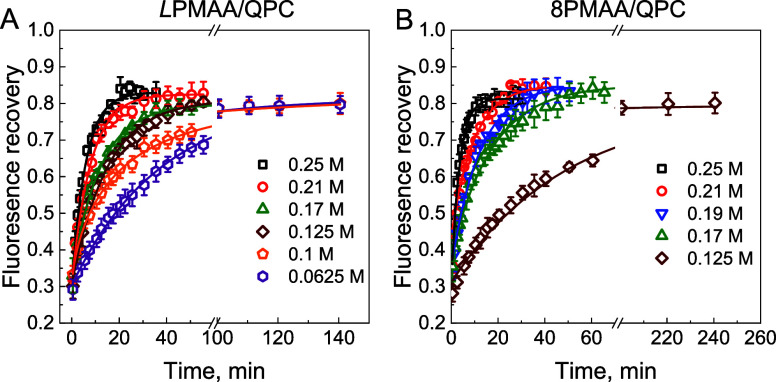
(A) Fluorescence recovery after photobleaching of (QPC/*L*PMAA)_2_/(QPC/*L*PMAA*)_3_/(QPC/*L*PMAA)_2_ (A) and (QPC/8PMAA)_2_/(QPC/8PMAA*)_3_/(QPC/8PMAA)_2_ (B) films deposited at pH 6 in 0.01 M phosphate buffer, bleached at 0.1 mW and monitored at 1 μW when exposed to 0.0625 M (hexagons), 0.1 M (pentagons), 0.125 M (diamonds), 0.17 M (up triangles), 0.19 M (down triangles), 0.21 M (circles), and 0.25 M (squares) NaCl solutions. The solid lines represent the exponential fit to the data.

We explored the lateral diffusion (*D*_*∥*_) of star polyelectrolytes using FRAP. These experiments were performed using fluorescently labeled polyacids (*L*PMAA*, 4PMAA*, 6PMAA*, and 8PMAA*), which were obtained by covalent modification of *L*PMAA, 4PMAA, 6PMAA, and 8PMAA with a fluorescent dye Alexa 488 (all the polymers contained one label per 800–1000 PMAA units, see Materials and Methods and Figure S12 for the labeling procedure and proof of reliable tethering of the label to polymer chains). The fluorescently labeled polyacids were deposited in the middle of the film ((BPEI/XPMAA/(QPC/XPMAA)_2_/(QPC/XPMAA*)_3_/(QPC/XPMAA)_2_) to avoid interfacial effects at the substrate and surface/water interfaces.^25,55^ The films were then exposed to solutions at different concentrations, and a spot of ∼0.28 μm in diameter was bleached with a 0.1 mW laser. The bleaching was performed to 25–30% of its initial fluorescent intensity to avoid possible crosslinking of polymer chains by the high-power laser.^24^ The laser intensity was then reduced to 1 μW, and recovery of the fluorescence intensity in the bleached area was recorded every 2–20 min. Figures 7 and S13 show the fluorescence recovery curves for films containing labeled linear and star polymers. Note that fluorescence intensity did not recover to initial values due to possible crosslinking between polymer chains during photobleaching.^24,56^ A strong dependence of the diffusivity of assembled PMAAs on salt concentration is evident from the data. The fluorescence recovery was fitted using an exponential function *I* = *I*_0_ + *A* × exp(−*t*/ τ), where *I*_0_ is fluorescence intensity recovered after reaching equilibrium, *I* is fluorescence intensity at time *t*, *A* is amplitude, and τ is recovery time. The half-recovery time of fluorescence intensity was determined as . The lateral diffusion coefficients were then calculated using the following equation:
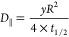
1where *y* is a constant beam shape factor (in our experiments 0.88),^25^*R* is the radius of the bleached spot (0.28 μm), and the half-recovery time.

**Figure 8:**
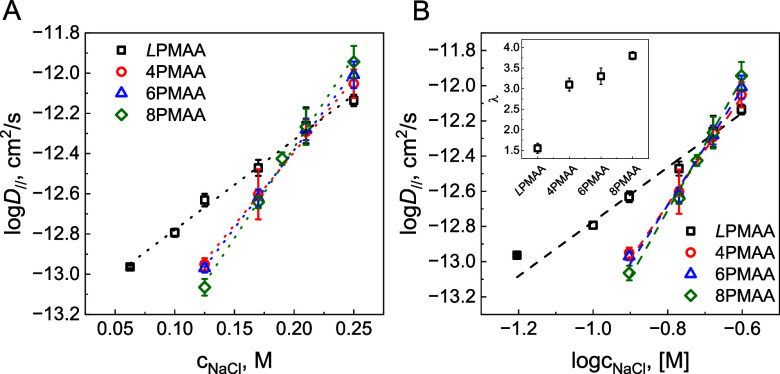
Salt dependences of lateral diffusion coefficients of the polyacids within (QPC/XPMAA)_2_/(QPC/XPMAA*)_3_/(QPC/XPMAA)_2_ films constructed using *L*PMAA* (squares), 4PMAA* (circles), 6PMAA* (triangles), and 8PMAA* (diamonds). The dotted lines in A represent semilogarithmic fits to the data (*R*^2^ ∼ 0.99), and dashed lines B were fitted using eq 2 (*R*^2^ ∼ 0.97–0.99). The inset represents the dependences of the length of the diffusing segment as a function of PMAA branching.

Figure 8 and Table S22 present the lateral diffusion coefficients of linear and star PMAA as a function of salt concentration. The most striking result is the greater sensitivity of the diffusivity of star polyacids to salt and an inversion of the trends of lateral diffusivity with the salt concentration for linear and star polyanions. Clearly, polymer topology determines the mechanism of diffusivity—star polymers feature a characteristic mechanism of diffusion in the condensed phase known as arm retraction.^57^ LbL films may be considered as condensed phases, enabling arm-retraction motion in star molecules. Within electrostatic assemblies, though, the diffusion of star polyacids should also be affected by electrostatic pairing with partner polyelectrolytes. However, despite lower ionization (Figure 4) and thus more sparse electrostatic pairing, the assembled star polymers were less diffusive than linear polymers at low salt concentrations (Figure 8). It is possible that this effect is due to slower dynamics of the core segments of star polymers^58^ affecting the molecular diffusivity of ionically assembled stars. At the same time, a stronger dependence of the diffusivity of star polymers on salt concentration reflects the high topological connectivity of molecular segments and eventually leads to higher diffusivity of star polymers at high salt concentrations. This result reflects distinct molecular mechanisms of diffusion and differences in the ionic pairing of linear and star polyacids within multilayer assemblies and is discussed in greater detail below.

Several theories have been developed to describe the effect of salt on the dynamics of polyelectrolytes included within LbL films and polymer coacervates.^18,21,59−62^Figure 8A shows the diffusion data plotted on a semilogarithmic scale, suggesting an exponential dependence of diffusion coefficients for the assembled polyacids on salt concentration. Semilogarithmic relationships of polyanion diffusion coefficients and salt concentration in LbL films have been observed in other studies for both lateral and perpendicular diffusion.^21,22,24^ For example, an AFM study of lateral diffusion in PSS/PDADMA systems exhibited a semilogarithmic dependence of the half-time of morphology smothering as a function of salt concentration.^21^ A model proposed in that study suggested that salt ions break polymer-polymer ionic contacts in a probabilistic manner and free up polymer segments for further rearrangement.^21^ Later, Selin et al. showed that lateral diffusion of PMAA in PMAA/QPC films of linear polymers deposited at pH 4.5 scaled exponentially with salt as *D*_*∥*_ =4 × 10^–14^e^2.6 × c_NaCl_^ cm^2^/s, following a dependence similar to that found for the *L*PMAA/QPC system here (*D*_*∥*_ =6 × 10^–14^e^4.5 × c_NaCl_^ cm^2^/s),^24^ with differences in response to salt attributed to the higher pH used in our experiments. Note that diffusion perpendicular to the substrate showed weaker responses to salt annealing for both the PMAA/QPC (*D*_⊥_ = 1.6 × 10^–17^e^1.9 × c_NaCl_^ cm^2^/s) and the PSS/PDADMA systems (*D*_*⊥*_ = 1.3 × 10^–16^e^1.8 × c_NaCl_^ cm^2^/s at 40 °C)^22^ due to the intrinsic anisotropy of polyelectrolyte diffusion within LbL films.^25^ Note that fitting of the data for star-containing system yielded *D*_*∥*_ =7 × 10^–15^e^9 × c_NaCl_^ cm^2^/s (for 8PMAA/QPC) showing that star polyacids are up to 2-fold more sensitive to changes in salt concentration relative to *L*PMAA.

Schlenoff et al. proposed another theory for polymer diffusion within LbL films based on “doping” of LbL films by salt ions and diffusion (hopping) of ionic pairs.^18,59^ Salt is considered to accelerate diffusion of polyelectrolytes via disruption of polymer-polymer ionic pairs and an increase in the proportion of monomers featuring extrinsically compensated charge. Polyelectrolyte diffusion occurs due to motions of polymer segments composed of more than one polymer unit so the diffusion coefficient scales with salt concentration to the power λ:
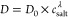
2where *D*_0_ is a proportionality constant and λ is the “length” of the diffusing polyelectrolyte segment expressed as the number of charged polymer units.

Figure 8B shows that our FRAP data could be successfully fitted with a logarithmic form of the power-law eq 2, with λ systematically increasing from 1.6 ± 0.1 for *L*PMAA to 4.0 ± 0.3 for 8PMAA. The value of λ for *L*PMAA (1.6 ± 0.1) is similar to that previously found in our FRAP experiments with linear PMAA/QPC (1.7 ± 0.4) deposited at pH 4.5.^24^ Formally, higher λ for star PMAA can indicate an increased “stiffness” of star PMAAs relative to linear chains. For example, values of λ close to 4 were reported for films of linear PSS/PDADMA polymers, where both polymer chains were stiffer than the PMAA used in this work.^18^ The argument of enhanced stiffness was employed to explain the solution behavior of 4- and 6-arm PAA polymers,^63^ though for nonlinear polymers of the same chemical makeup, λ likely reflects differences in topological connectivity rather than chemical-composition-dictated chain stiffness. In addition, the proportionality constant *D*_0_ increased with polyacid branching (Table 3), suggesting easier salt doping in films of star polymers,^18^ in good agreement with the greater swelling of star-containing LbL films in NaCl solutions seen in Figure 2B.

**Table 3:** Effect of Polymer Architecture on “Length” of Diffusing Polyelectrolyte Segment λ and a Proportionality Constant *D*_0_

	λ	log *D*_0_
*L*PMAA	1.6 ± 0.1	(−11.2 ± 0.1)
4PMAA	3.1 ± 0.1	(−10.2 ± 0.1)
6PMAA	3.4 ± 0.2	(−10 ± 0.1)
8PMAA	4.0 ± 0.2	(−9.7 ± 0.1)

The dynamics of polyelectrolytes has also been explored for polymer coacervates and treated using the Rubinstein and Semenov theory of “sticky reptation.”^60,64^ In particular, Spruijt et al. suggested that the activation energy of ionic pairing in polyelectrolyte complexes is dependent on the electrical free energy of four separated ionic groups of charge ± e in solution and the Coulombic energy of two ionic pairs in contact.^60^ This model, with some modifications, was later used to describe dynamics in several polymer coacervate systems.^61,62,65−68^ In particular, Hamad et al.^62^ proposed that diffusion of polyelectrolytes within coacervates involves association/dissociation of several consecutive ionic pairs (*n*), and the pair lifetime can be calculated as:
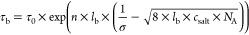
3where τ_0_ is a proportionality constant (s^–1^), *l*_b_ is Bjerrum length (m), σ is a contact distance (m), and *N*_A_ is the Avogadro’s number (mol^–1^). Equation 3 can be written as:
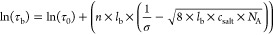
4

**Figure 9:**
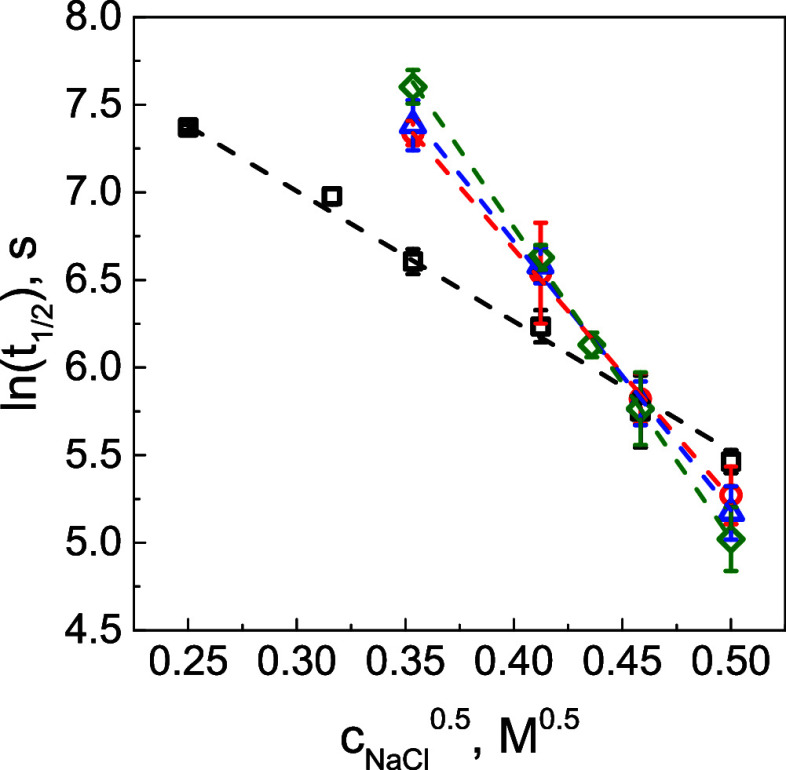
Half-recovery time as a function of square root salt concentration for *L*PMAA*- (squares), 4PMAA* (circles), 6PMAA* (triangles), and 8PMAA*-containing (diamonds) films. The dashed lines represent linear fits to the data using the eq 4.

Figure 9 shows fits of our half-recovery time data as a function of the square root of salt concentration. Assuming that the half-recovery time in FRAP experiments is proportional to the ionic pair lifetime and using eq 4 with Bjerrum length of 0.71 nm (as in water), a contact distance σ equal to 0.484 nm (assuming the same effective ionic radius of the QPC/PMAA ionic pair using 0.162 nm ionic radius of acetate ion^69^ and 0.322 nm ionic radius of tetra(methyl) ammonium ion^70^), values n can be calculated from the fitted data. In the case of the *L*PMAA/QPC system, *n* was 3.2 ± 0.1, while it was 6.1 ± 0.1, 6.6 ± 0.2, and 7.7 ± 0.2 for 4PMAA/QPC, 6PMAA/QPC, and 8PMAA/QPC films, respectively. Note that these numbers are twice larger than those of the diffusing segment in Schlenoff’s hopping model in Table 3 (3.2 vs 1.6 for *L*PMAA and 7.7 vs 4.0 for 8PMAA). The difference between these models probably arises from the fact that when fitting the data with eq 4, a Bjerrum length of 0.71 for water was assumed. However, earlier studies have revealed that the dielectric environment within LbL films is different, featuring dielectric constants (ε_r_) significantly lower than for water.^71−74^ Assuming that n in the Hamad-Colby model is the same as λ in the ion-hopping model and using these values to fit the data in Figure 9 yields a Bjerrum length of 1.13 ± 0.01 nm and ε_r_ of 49.5 ± 0.5. The obtained dielectric constant is similar to ε_r_ = 45, which was suggested for poly(acrylic acid)/(poly(*N*,*N*-dimethylaminoethyl methacrylate) coacervate by Spruijt et al.,^60^ and ε_r_ = 50 calculated from the fluorescence measurements for the PSS/poly(allylamine hydrochloride) LbL films.^71^ Syed and Srivastava suggested that counterions accompanying the polyelectrolytes in coacervates play a significant role in polymer dynamics.^68^ Thus, we recalculated the apparent salt concentration in the swollen film (see Figure S14 caption) based on the swelling data from Figure 2B and refitted that data using eq 4 (Figure S14). While *n* slightly decreased (from 3.2 to 2.5 for *L*PMAA and from 7.7 to 5.7 for 8PMAA), the general trend remained the same, including a greater sensitivity of star-containing films to salt and the inversion of the diffusivity of linear and star polymers in low and high salt concentration regimes.

In conclusion, here we have explored the diffusion of polyelectrolytes within LbL assemblies of weak polyacids of matched molecular weight but varied molecular architecture and found that star-containing films exhibit several distinct properties relative to their assembled linear counterparts, such as a lower number of ionic contacts, leading to greater swelling and lower salt stability in star-containing films. While the swelling and salt stability results can be broadly understood in terms of greater compactness and segmental crowding at the core of star polymers resulting in more “particle-like” behavior,^75^ this work quantifies the consequences of such compactness for assembled weak polyelectrolyte polyacids, which are ionically paired with linear counterparts in LbL films. We have demonstrated that the star architecture results in lower ionization and a lesser degree of ionic pairing of weak star polyelectrolytes in LbL assemblies, resulting from both their branched topology and an increased local segmental density of ionizable groups.

Perhaps the most significant result of this work is the demonstration of an unusual crossover in the diffusivity rate between linear and star polyelectrolytes in low-salt and high-salt regimes. Despite greater compactness and lower density of ionic pairs constraining molecular diffusivity, star polyelectrolytes exhibited lower lateral diffusivity in LbL films as compared with linear chains in the low-salt regime. We believe that this reflects a topology-induced slowing of segmental motions of the star polymers due to crowding at the star core region. At the same time, greater topological connectivity of star polymers resulted in a stronger, more cooperative response of LbL assemblies to increasing salt concentrations, manifested in greater molecular diffusivity of star polyelectrolytes at high salt concentrations.

In this work, the results on molecular diffusivity were compared with previously developed theories of polyelectrolyte mobility in LbL films and coacervates, giving good agreement and all showing an increase in the number of simultaneously diffusing units with increasing polymer branching. The stronger response of star polymers to changes in salt concentration likely indicates a distinct mechanism of diffusion for branched macromolecules in LbL films, possibly resembling diffusion of stars in condensed phases that occurs via the arm-retraction mechanism.^57^ However, the contribution of the electrostatic pairing of the star arms to molecular diffusion within LbL films and polyelectrolyte coacervates should be explored experimentally and theoretically. Finally, we note that the magnitude of the diffusion coefficients for assembled polyelectrolyte chains in the directions perpendicular and parallel to the surface remain largely distinct, with an approximately 2 × 10^3^ difference between the all-linear system and ∼8 × 10^2^ for the star-containing system at 0.25 M NaCl, suggesting a stronger anisotropy of diffusion of linear chains within LbL films and indirectly supporting the different underlying mechanisms of diffusion of the polyacids of different molecular architecture.

## Supporting Information Available

Kinetics of polymerization of linear and star polymers, NMR data of PTBMA and PMAA, kinetics of PMAA/QPC swelling and film decomposition, raw neutron reflectivity data, corresponding neutron scattering length density profiles, model parameters for PMAA/QPC films, as well as FRAP recovery curves for 4PMAA/QPC and 6PMAA/QPC films (PDF)

## References

[ref1] Park S.; Han U.; Choi D.; Hong J. Layer-by-layer assembled polymeric thin films as prospective drug delivery carriers: design and applications. Biomater. Res. 2018, 22, 29 10.1186/s40824-018-0139-5. 30275972 PMC6158909

[ref2] Kozlovskaya V.; Dolmat M.; Kharlampieva E. Two-Dimensional and Three-Dimensional Ultrathin Multilayer Hydrogels through Layer-by-Layer Assembly. Langmuir 2022, 38, 7867–7888. 10.1021/acs.langmuir.2c00630. 35686955

[ref3] Greenspon A. S.; Marceaux B. L.; Hu E. L. Robust lanthanide emitters in polyelectrolyte thin films for photonic applications. Nanotechnology 2018, 29, 075302 10.1088/1361-6528/aaa325. 29260734

[ref4] Brito J.; Asawa K.; Marin A.; Andrianov A. K.; Choi C.-H.; Sukhishvili S. A. Hierarchically Structured, All-Aqueous-Coated Hydrophobic Surfaces with pH-Selective Droplet Transfer Capability. ACS Appl. Mater. Interfaces 2022, 14, 26225–26237. 10.1021/acsami.2c04499. 35611942

[ref5] Zhou J.; Ellis A. V.; Voelcker N. H. Recent developments in PDMS surface modification for microfluidic devices. Electrophoresis 2010, 31, 2–16. 10.1002/elps.200900475. 20039289

[ref6] Wang Z.; Ouyang L.; Li H.; Wågberg L.; Hamedi M. M. Layer-by-Layer Assembly of Strong Thin Films with High Lithium Ion Conductance for Batteries and Beyond. Small 2021, 17, 2100954 10.1002/smll.202100954. 34212496

[ref7] Tolganbek N.; Mentbayeva A.; Serik N.; Batyrgali N.; Naizakarayev M.; Kanamura K.; Bakenov Z. Design and preparation of thin film gel polymer electrolyte for 3D Li-ion battery. J. Power Sources 2021, 493, 229686 10.1016/j.jpowsour.2021.229686.

[ref8] Mentbayeva A.; Sukhishvili S.; Naizakarayev M.; Batyrgali N.; Seitzhan Z.; Bakenov Z. Ultrathin clay-containing layer-by-layer separator coating enhances performance of lithium-sulfur batteries. Electrochim. Acta 2021, 366, 137454 10.1016/j.electacta.2020.137454.

[ref9] Soltwedel O.; Ivanova O.; Nestler P.; Müller M.; Köhler R.; Helm C. A. Interdiffusion in Polyelectrolyte Multilayers. Macromolecules 2010, 43, 7288–7293. 10.1021/ma101279q.

[ref10] Xu L.; Ankner J. F.; Sukhishvili S. A. Steric Effects in Ionic Pairing and Polyelectrolyte Interdiffusion within Multilayered Films: A Neutron Reflectometry Study. Macromolecules 2011, 44, 6518–6524. 10.1021/ma200986d.

[ref11] Xu L.; Pristinski D.; Zhuk A.; Stoddart C.; Ankner J. F.; Sukhishvili S. A. Linear versus Exponential Growth of Weak Polyelectrolyte Multilayers: Correlation with Polyelectrolyte Complexes. Macromolecules 2012, 45, 3892–3901. 10.1021/ma300157p.

[ref12] Sukhishvili S. A.; Kharlampieva E.; Izumrudov V. Where Polyelectrolyte Multilayers and Polyelectrolyte Complexes Meet. Macromolecules 2006, 39, 8873–8881. 10.1021/ma061617p.

[ref13] Xu L.; Selin V.; Zhuk A.; Ankner J. F.; Sukhishvili S. A. Molecular Weight Dependence of Polymer Chain Mobility within Multilayer Films. ACS Macro Lett. 2013, 2, 865–868. 10.1021/mz400413v. 35607005

[ref14] Nestler P.; Paßvogel M.; Helm C. A. Influence of Polymer Molecular Weight on the Parabolic and Linear Growth Regime of PDADMAC/PSS Multilayers. Macromolecules 2013, 46, 5622–5629. 10.1021/ma400333f.

[ref15] Abbett R. L.; Chen Y.; Schlenoff J. B. Self-Exchange of Polyelectrolyte in Multilayers: Diffusion as a Function of Salt Concentration and Temperature. Macromolecules 2021, 54, 9522–9531. 10.1021/acs.macromol.1c01464.

[ref16] Sill A.; Nestler P.; Azinfar A.; Helm C. A. Tailorable Polyanion Diffusion Coefficient in LbL Films: The Role of Polycation Molecular Weight and Polymer Conformation. Macromolecules 2019, 52, 9045–9052. 10.1021/acs.macromol.9b01761.

[ref17] Lavalle P.; Voegel J.-C.; Vautier D.; Senger B.; Schaaf P.; Ball V. Dynamic Aspects of Films Prepared by a Sequential Deposition of Species: Perspectives for Smart and Responsive Materials. Adv. Mater. 2011, 23, 1191–1221. 10.1002/adma.201003309. 21264957

[ref18] Jomaa H. W.; Schlenoff J. B. Salt-Induced Polyelectrolyte Interdiffusion in Multilayered Films: A Neutron Reflectivity Study. Macromolecules 2005, 38, 8473–8480. 10.1021/ma050072g.

[ref19] Dubas S. T.; Schlenoff J. B. Swelling and Smoothing of Polyelectrolyte Multilayers by Salt. Langmuir 2001, 17, 7725–7727. 10.1021/la0112099.

[ref20] Fares H. M.; Schlenoff J. B. Diffusion of Sites versus Polymers in Polyelectrolyte Complexes and Multilayers. J. Am. Chem. Soc. 2017, 139, 14656–14667. 10.1021/jacs.7b07905. 28981268

[ref21] McAloney R. A.; Dudnik V.; Goh M. C. Kinetics of Salt-Induced Annealing of a Polyelectrolyte Multilayer Film Morphology. Langmuir 2003, 19, 3947–3952. 10.1021/la026882s.

[ref22] Sill A.; Nestler P.; Thran P.; Helm C. A. Dependence of PSS Diffusion in Multilayers of Entangled PDADMA on Temperature and Salt Concentration: More than One Diffusion Constant. Macromolecules 2021, 54, 9372–9384. 10.1021/acs.macromol.1c00639.

[ref23] Zhuk A.; Selin V.; Zhuk I.; Belov B.; Ankner J. F.; Sukhishvili S. A. Chain Conformation and Dynamics in Spin-Assisted Weak Polyelectrolyte Multilayers. Langmuir 2015, 31, 3889–3896. 10.1021/acs.langmuir.5b00401. 25768113

[ref24] Selin V.; Ankner J. F.; Sukhishvili S. A. Diffusional Response of Layer-by-Layer Assembled Polyelectrolyte Chains to Salt Annealing. Macromolecules 2015, 48, 3983–3990. 10.1021/acs.macromol.5b00361.

[ref25] Xu L.; Kozlovskaya V.; Kharlampieva E.; Ankner J. F.; Sukhishvili S. A. Anisotropic Diffusion of Polyelectrolyte Chains within Multilayer Films. ACS Macro Lett. 2012, 1, 127–130. 10.1021/mz200075x. PMC3454581 23019538

[ref26] Ren J. M.; McKenzie T. G.; Fu Q.; Wong E. H. H.; Xu J.; An Z.; Shanmugam S.; Davis T. P.; Boyer C.; Qiao G. G. Star Polymers. Chem. Rev. 2016, 116, 6743–6836. 10.1021/acs.chemrev.6b00008. 27299693

[ref27] Cosimbescu L.; Robinson J. W.; Zhou Y.; Qu J. Dual functional star polymers for lubricants. RSC Adv. 2016, 6, 86259–86268. 10.1039/C6RA17461B.

[ref28] van Ravensteijn B. G. P.; Bou Zerdan R.; Seo D.; Cadirov N.; Watanabe T.; Gerbec J. A.; Hawker C. J.; Israelachvili J. N.; Helgeson M. E. Triple Function Lubricant Additives Based on Organic–Inorganic Hybrid Star Polymers: Friction Reduction, Wear Protection, and Viscosity Modification. ACS Appl. Mater. Interfaces 2019, 11, 1363–1375. 10.1021/acsami.8b16849. 30525414

[ref29] Giuntoli A.; Hansoge N. K.; Keten S. Star topology increases ballistic resistance in thin polymer films. Extreme Mech. Lett. 2020, 41, 101038 10.1016/j.eml.2020.101038.

[ref30] Giuntoli A.; Keten S. Tuning star architecture to control mechanical properties and impact resistance of polymer thin films. Cell Rep. Phys. Sci. 2021, 2, 100596 10.1016/j.xcrp.2021.100596.

[ref31] Gao H. Development of Star Polymers as Unimolecular Containers for Nanomaterials. Macromol. Rapid Commun. 2012, 33, 722–734. 10.1002/marc.201200005. 22419360

[ref32] Wiltshire J. T.; Qiao G. G. Recent Advances in Star Polymer Design: Degradability and the Potential for Drug Delivery. Aust. J. Chem. 2007, 60, 699–705. 10.1071/CH07128.

[ref33] Zhang Y.; Yan J.; Avellan A.; Gao X.; Matyjaszewski K.; Tilton R. D.; Lowry G. V. Temperature- and pH-Responsive Star Polymers as Nanocarriers with Potential for in Vivo Agrochemical Delivery. ACS Nano 2020, 14, 10954–10965. 10.1021/acsnano.0c03140. 32628009

[ref34] Georgiou T. K. Star polymers for gene delivery. Polym. Int. 2014, 63, 1130–1133. 10.1002/pi.4718.

[ref35] Xu W.; Ledin P. A.; Shevchenko V. V.; Tsukruk V. V. Architecture, Assembly, and Emerging Applications of Branched Functional Polyelectrolytes and Poly(ionic liquid)s. ACS Appl. Mater. Interfaces 2015, 7, 12570–12596. 10.1021/acsami.5b01833. 26010902

[ref36] Connal L. A.; Li Q.; Quinn J. F.; Tjipto E.; Caruso F.; Qiao G. G. pH-Responsive Poly(acrylic acid) Core Cross-Linked Star Polymers: Morphology Transitions in Solution and Multilayer Thin Films. Macromolecules 2008, 41, 2620–2626. 10.1021/ma7019557.

[ref37] Kim B.-S.; Gao H.; Argun A. A.; Matyjaszewski K.; Hammond P. T. All-Star Polymer Multilayers as pH-Responsive Nanofilms. Macromolecules 2009, 42, 368–375. 10.1021/ma801812v.

[ref38] Guo Z.; Chen X.; Xin J.; Wu D.; Li J.; Xu C. Effect of Molecular Weight and Arm Number on the Growth and pH-Dependent Morphology of Star Poly[2-(dimethylamino)ethyl methacrylate]/Poly(styrenesulfonate) Multilayer Films. Macromolecules 2010, 43, 9087–9093. 10.1021/ma1013429.

[ref39] Choi I.; Suntivich R.; Plamper F. A.; Synatschke C. V.; Müller A. H. E.; Tsukruk V. V. pH-Controlled Exponential and Linear Growing Modes of Layer-by-Layer Assemblies of Star Polyelectrolytes. J. Am. Chem. Soc. 2011, 133, 9592–9606. 10.1021/ja203106c. 21591785

[ref40] Chen F.; Liu G.; Zhang G. Formation of Multilayers by Star Polyelectrolytes: Effect of Number of Arms on Chain Interpenetration. J. Phys. Chem. B 2012, 116, 10941–10950. 10.1021/jp304994k. 22861067

[ref41] Aliakseyeu A.; Ankner J. F.; Sukhishvili S. A. Impact of Star Polyacid Branching on Polymer Diffusion within Multilayer Films. Macromolecules 2022, 55, 8150–8161. 10.1021/acs.macromol.2c01104.

[ref42] Aliakseyeu A.; Hlushko R.; Sukhishvili S. A. Nonionic star polymers with upper critical solution temperature in aqueous solutions. Polym. Chem. 2022, 13, 2637–2650. 10.1039/D2PY00216G.

[ref43] Ramirez R.; Woodcock J.; Kilbey S. M. ARGET-ATRP synthesis and swelling response of compositionally varied poly(methacrylic acid-co-N,N-diethylaminoethyl methacrylate) polyampholyte brushes. Soft Matter 2018, 14, 6290–6302. 10.1039/C8SM00882E. 30014055

[ref44] Selin V.; Ankner J. F.; Sukhishvili S. A. Nonlinear Layer-by-Layer Films: Effects of Chain Diffusivity on Film Structure and Swelling. Macromolecules 2017, 50, 6192–6201. 10.1021/acs.macromol.7b01218.

[ref45] Aliakseyeu A.; Albright V.; Yarbrough D.; Hernandez S.; Zhou Q.; Ankner J. F.; Sukhishvili S. A. Selective hydrogen bonding controls temperature response of layer-by-layer upper critical solution temperature micellar assemblies. Soft Matter 2021, 17, 2181–2190. 10.1039/D0SM01997F. 33458733

[ref46] Kozlovskaya V.; Stockmal K. A.; Higgins W.; Ankner J. F.; Morgan S. E.; Kharlampieva E. Architecture of Hydrated Multilayer Poly(methacrylic acid) Hydrogels: The Effect of Solution pH. ACS Appl. Polym. Mater. 2020, 2, 2260–2273. 10.1021/acsapm.0c00240.

[ref47] Pristinski D.; Kozlovskaya V.; Sukhishvili S. A. Fluorescence correlation spectroscopy studies of diffusion of a weak polyelectrolyte in aqueous solutions. J. Chem. Phys. 2005, 122, 014907 10.1063/1.1829255. 15638700

[ref48] Petrásek Z.; Schwille P. Precise measurement of diffusion coefficients using scanning fluorescence correlation spectroscopy. Biophys. J. 2008, 94, 1437–1448. 10.1529/biophysj.107.108811. 17933881 PMC2212689

[ref49] Jakubowski W.; Matyjaszewski K. Activators Regenerated by Electron Transfer for Atom-Transfer Radical Polymerization of (Meth)acrylates and Related Block Copolymers. Angew. Chem., Int. Ed. 2006, 45, 4482–4486. 10.1002/anie.200600272. 16770821

[ref50] Matyjaszewski K.; Jakubowski W.; Min K.; Tang W.; Huang J.; Braunecker W. A.; Tsarevsky N. V. Diminishing catalyst concentration in atom transfer radical polymerization with reducing agents. Proc. Natl. Acad. Sci. 2006, 103, 15309–15314. 10.1073/pnas.0602675103. 17032773 PMC1622823

[ref51] Saigal T.; Riley J. K.; Golas P. L.; Bodvik R.; Claesson P. M.; Matyjaszewski K.; Tilton R. D. Poly(Ethylene Oxide) Star Polymer Adsorption at the Silica/Aqueous Interface and Displacement by Linear Poly(Ethylene Oxide). Langmuir 2013, 29, 3999–4007. 10.1021/la305085a. 23448185

[ref52] Tanchak O. M.; Barrett C. J. Swelling Dynamics of Multilayer Films of Weak Polyelectrolytes. Chem. Mater. 2004, 16, 2734–2739. 10.1021/cm049920x.

[ref53] Choi J.; Rubner M. F. Influence of the Degree of Ionization on Weak Polyelectrolyte Multilayer Assembly. Macromolecules 2005, 38, 116–124. 10.1021/ma048596o.

[ref54] Nestler P.; Paßvogel M.; Ahrens H.; Soltwedel O.; Köhler R.; Helm C. A. Branched Poly(ethylenimine) as Barrier Layer for Polyelectrolyte Diffusion in Multilayer Films. Macromolecules 2015, 48, 8546–8556. 10.1021/acs.macromol.5b01065.

[ref55] Nazaran P.; Bosio V.; Jaeger W.; Anghel D. F.; Klitzing R. v. Lateral Mobility of Polyelectrolyte Chains in Multilayers. J. Phys. Chem. B 2007, 111, 8572–8581. 10.1021/jp068768e. 17461569

[ref56] Reits E. A. J.; Neefjes J. J. From fixed to FRAP: measuring protein mobility and activity in living cells. Nat. Cell Biol. 2001, 3, E145–E147. 10.1038/35078615. 11389456

[ref57] Frischknecht A. L.; Milner S. T. Self-Diffusion with Dynamic Dilution in Star Polymer Melts. Macromolecules 2000, 33, 9764–9768. 10.1021/ma000918a.

[ref58] Zhang W.; Douglas J. F.; Chremos A.; Starr F. W. Structure and Dynamics of Star Polymer Films from Coarse-Grained Molecular Simulations. Macromolecules 2021, 54, 5344–5353. 10.1021/acs.macromol.1c00504.

[ref59] Farhat T. R.; Schlenoff J. B. Doping-Controlled Ion Diffusion in Polyelectrolyte Multilayers: Mass Transport in Reluctant Exchangers. J. Am. Chem. Soc. 2003, 125, 4627–4636. 10.1021/ja021448y. 12683835

[ref60] Spruijt E.; Sprakel J.; Lemmers M.; Stuart M. A. C.; van der Gucht J. Relaxation Dynamics at Different Time Scales in Electrostatic Complexes: Time-Salt Superposition. Phys. Rev. Lett. 2010, 105, 208301 10.1103/PhysRevLett.105.208301. 21231268

[ref61] Yang M.; Shi J.; Schlenoff J. B. Control of Dynamics in Polyelectrolyte Complexes by Temperature and Salt. Macromolecules 2019, 52, 1930–1941. 10.1021/acs.macromol.8b02577.

[ref62] Hamad F. G.; Chen Q.; Colby R. H. Linear Viscoelasticity and Swelling of Polyelectrolyte Complex Coacervates. Macromolecules 2018, 51, 5547–5555. 10.1021/acs.macromol.8b00401.

[ref63] Moinard D.; Borsali R.; Taton D.; Gnanou Y. Scattering and Viscosimetric Behaviors of Four- and Six-Arm Star Polyelectrolyte Solutions. Macromolecules 2005, 38, 7105–7120. 10.1021/ma050505f.

[ref64] Rubinstein M.; Semenov A. N. Dynamics of Entangled Solutions of Associating Polymers. Macromolecules 2001, 34, 1058–1068. 10.1021/ma0013049.

[ref65] Ali S.; Prabhu V. M. Relaxation Behavior by Time-Salt and Time-Temperature Superpositions of Polyelectrolyte Complexes from Coacervate to Precipitate. Gels 2018, 4, 11 10.3390/gels4010011. 30674787 PMC6318648

[ref66] Marciel A. B.; Srivastava S.; Tirrell M. V. Structure and rheology of polyelectrolyte complex coacervates. Soft Matter 2018, 14, 2454–2464. 10.1039/C7SM02041D. 29376531

[ref67] Shamoun R. F.; Hariri H. H.; Ghostine R. A.; Schlenoff J. B. Thermal Transformations in Extruded Saloplastic Polyelectrolyte Complexes. Macromolecules 2012, 45, 9759–9767. 10.1021/ma302075p.

[ref68] Syed V. M. S.; Srivastava S. Time–Ionic Strength Superposition: A Unified Description of Chain Relaxation Dynamics in Polyelectrolyte Complexes. ACS Macro Lett. 2020, 9, 1067–1073. 10.1021/acsmacrolett.0c00252. 35648617

[ref69] Zhang W.; Saliba M.; Moore D. T.; Pathak S. K.; Hörantner M. T.; Stergiopoulos T.; Stranks S. D.; Eperon G. E.; Alexander-Webber J. A.; Abate A.; Sadhanala A.; Yao S.; Chen Y.; Friend R. H.; Estroff L. A.; Wiesner U.; Snaith H. J. Ultrasmooth organic–inorganic perovskite thin-film formation and crystallization for efficient planar heterojunction solar cells. Nat. Commun. 2015, 6, 6142 10.1038/ncomms7142. 25635571

[ref70] Jin I. S.; Parida B.; Jung J. W. Simultaneously enhanced efficiency and ambient stability of inorganic perovskite solar cells by employing tetramethylammonium chloride additive in CsPbI2Br. J. Mater. Sci. Technol. 2022, 102, 224–231. 10.1016/j.jmst.2021.05.084.

[ref71] Tedeschi C.; Möhwald H.; Kirstein S. Polarity of Layer-by-Layer Deposited Polyelectrolyte Films As Determined by Pyrene Fluorescence. J. Am. Chem. Soc. 2001, 123, 954–960. 10.1021/ja0031974. 11456630

[ref72] Schönhoff M.; Ball V.; Bausch A. R.; Dejugnat C.; Delorme N.; Glinel K.; Klitzing R. v.; Steitz R. Hydration and internal properties of polyelectrolyte multilayers. Colloids Surf., A 2007, 303, 14–29. 10.1016/j.colsurfa.2007.02.054.

[ref73] Neff P. A.; Naji A.; Ecker C.; Nickel B.; Klitzing R. v.; Bausch A. R. Electrical Detection of Self-Assembled Polyelectrolyte Multilayers by a Thin Film Resistor. Macromolecules 2006, 39, 463–466. 10.1021/ma0519213.

[ref74] Neff P. A.; Wunderlich B. K.; Klitzing R. v.; Bausch A. R. Formation and Dielectric Properties of Polyelectrolyte Multilayers Studied by a Silicon-on-Insulator Based Thin Film Resistor. Langmuir 2007, 23, 4048–4052. 10.1021/la063632t. 17315907

[ref75] Chremos A.; Douglas J. F. Solution properties of star polyelectrolytes having a moderate number of arms. J. Chem. Phys. 2017, 147, 044906 10.1063/1.4995534. 28764357 PMC5702915

