# Supplementary material for: Salt-Induced Diffusion of Star and Linear Polyelectrolytes within Multilayer Films
Source: Macromolecules. 2023 Jul 10;56(14):5434–45. doi: 10.1021/acs.macromol.3c00777 (PMC10863069; doi:10.1021/acs.macromol.3c00777)
Supplement: Supplementary file 1 — ma3c00777_si_001.pdf [file ma3c00777_si_001.pdf]

Supporting Information

Salt-Induced Diffusion of Star and Linear Polyelectrolytes within Multilayer Films

Aliaksei Aliakseyeu[1], Parin Purvin Shah[1], John F. Ankner[2], Svetlana A. Sukhishvili[1]*

[1]*Department of Materials Science & Engineering, Texas A&M University,*

College Station, Texas 77843, USA

[2] *Spallation Neutron Source Second Target Station Project, Oak Ridge National Laboratory, Oak Ridge, Tennessee 37831, USA*

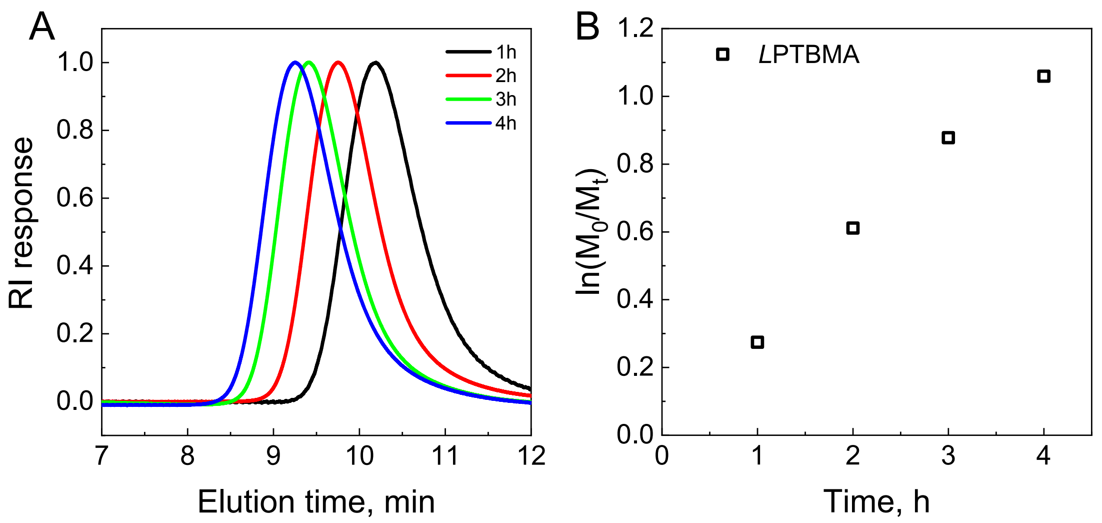

Fig. S1. ARGET ATRP polymerization of TBMA using a EtBiB ATRP initiator: (A) GPC traces at different polymerization times and (B) time evolution of $\ln[M]_0/[M]_t$ during synthesis of linear PTBMA.

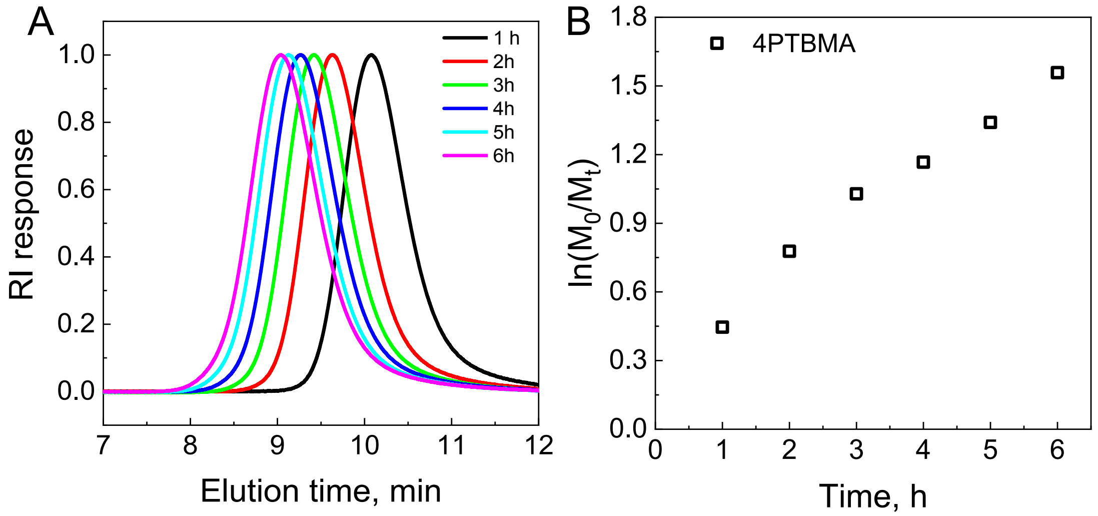

Fig. S2. ARGET ATRP polymerization of TBMA using 4f-BiB ATRP initiator: (A) GPC traces at different polymerization times and (B) time evolution of $\ln[M]_0/[M]_t$ during synthesis of 4-arm star PTBMA.

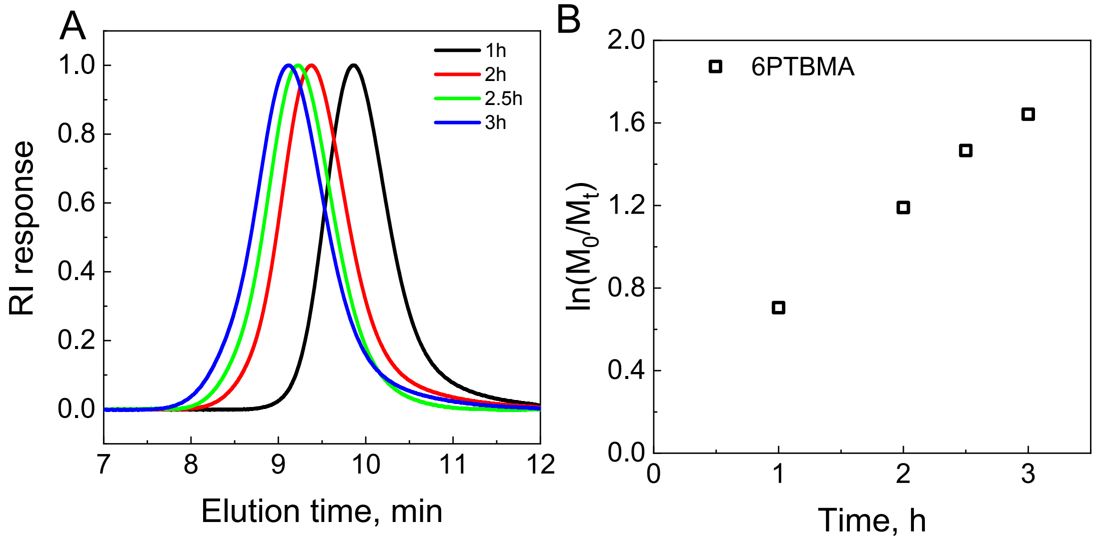

Fig. S3. ARGET ATRP polymerization of TBMA using 6f-BiB ATRP initiator: (A) GPC traces at different polymerization times and (B) time evolution of $\ln[M]_0/[M]_t$ during synthesis of 6-arm star PTBMA.

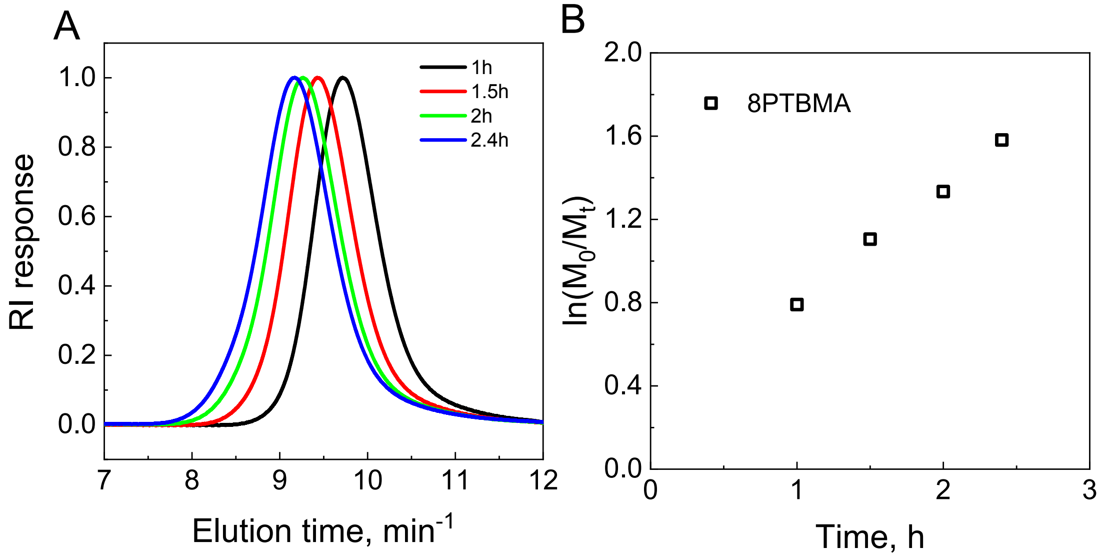

Fig. S4. ARGET ATRP polymerization of TBMA using 8f-BiB ATRP initiator: (A) GPC traces at different polymerization times and (B) time evolution of $\ln[M]_0/[M]_t$ during synthesis of 8-arm star PTBMA.

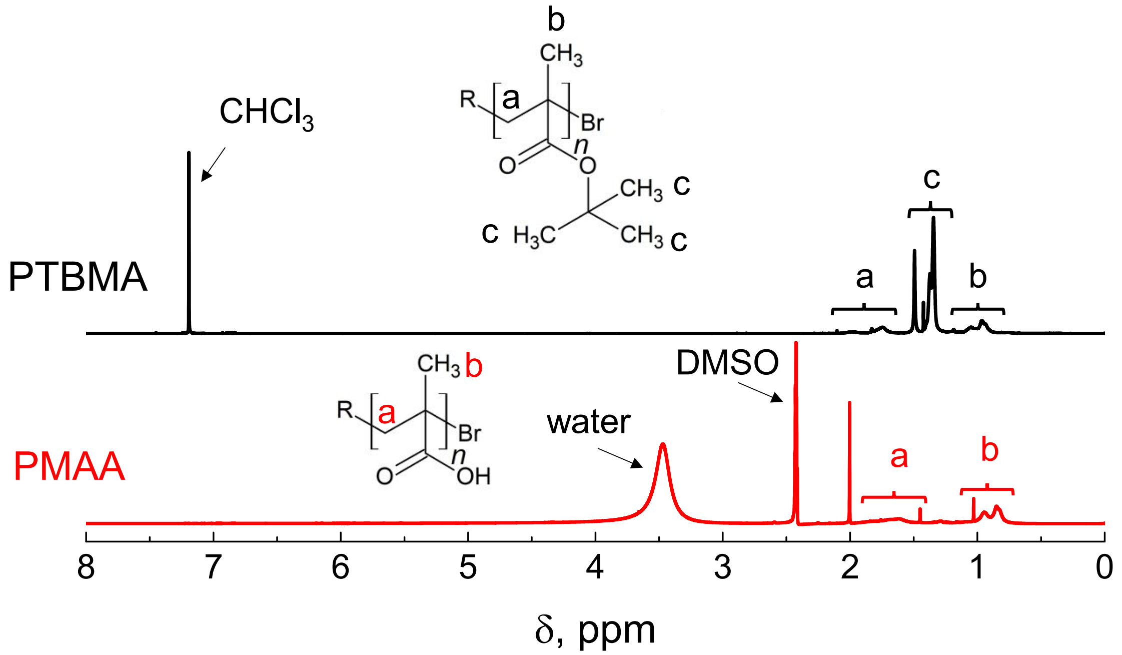

Fig. S5. ^{1}H NMR of linear PTBMA in deuterated chloroform, $CDCl_3$ (top) and deprotected linear PMAA in deuterated DMSO, $(CD_3)_2SO$ (bottom).

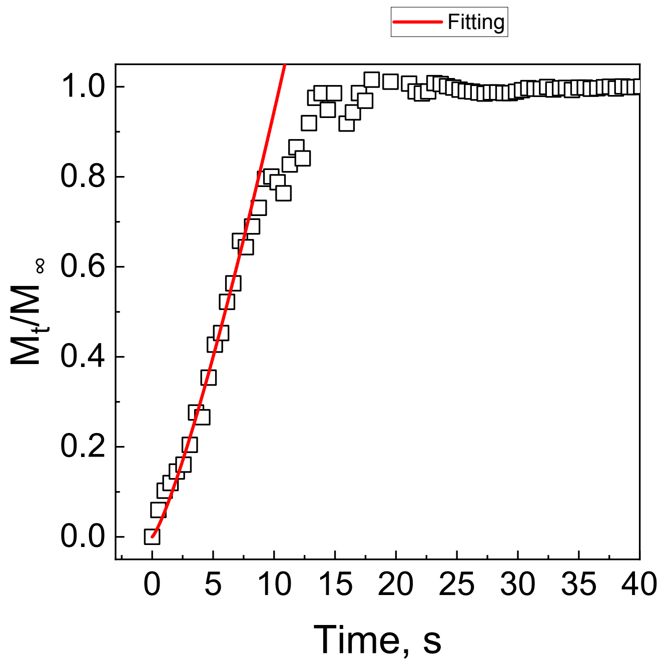

Fig. S6. An example of kinetics of swelling for $(QPC/6PMAA)_{20}$ in 0.15 M NaCl at pH 6. The red line represents the fit of the data using the equation $\frac{M_t}{M_\infty} = k_1 * t^n$, where M_t is increase of the film thickness at time t, M_∞ is increase of the film thickness at the equilibrium state, k_1 is a characteristic constant, t is time and n is the exponent that describes the diffusion type.[3] The fitting parameters are shown in Table S1.

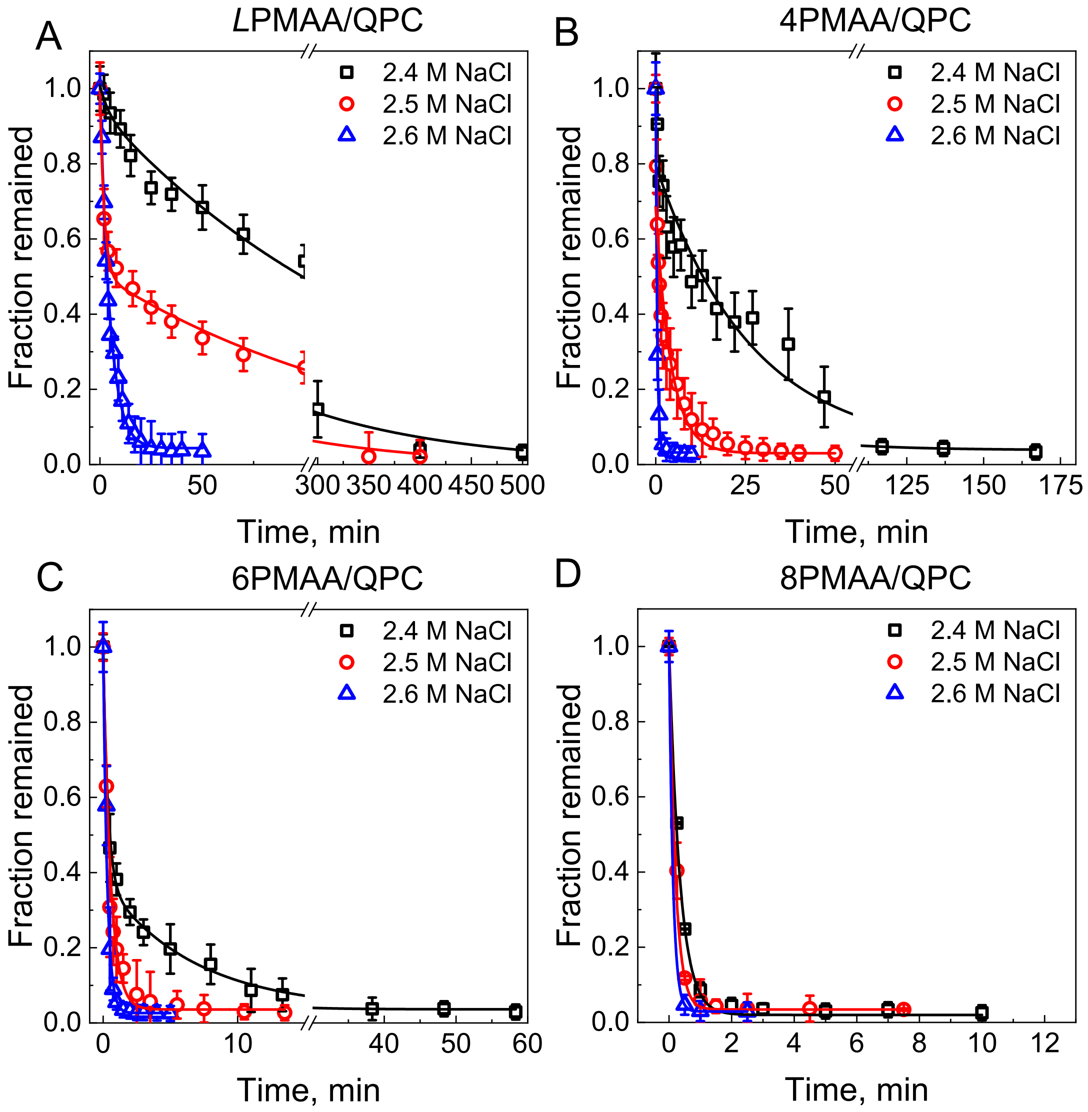

Fig. S7. Kinetics of (A) (LPMAA/QPC)$_{20}$, (B) (4PMAA/QPC)$_{20}$, (C) (6PMAA/QPC)$_{20}$, and (D) (8PMAA/QPC)$_{20}$ film erosion in 2.4 M (squares), 2.5 (open circles) and 2.6 M (triangles) NaCl solutions. The solid lines represent fitting to the data using the single exponential function.

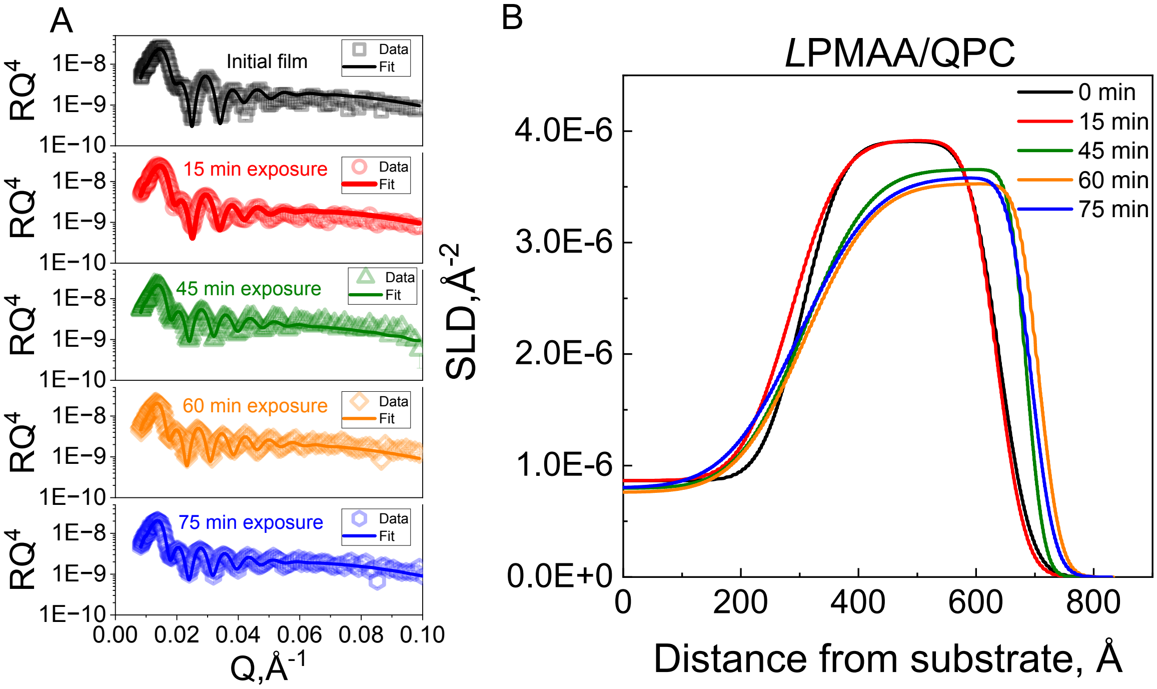

Fig. S8. (A) Neutron reflectivity data (plotted as RQ^4 to enhance small features) and (B) the corresponding neutron scattering length density profiles for *L*PMAA/(*h*QPC/*L*PMAA)$_4$/(*d*QPC/*L*PMAA)$_3$ LbL film deposited at pH 6 before and after exposure to 0.25 M NaCl solutions.

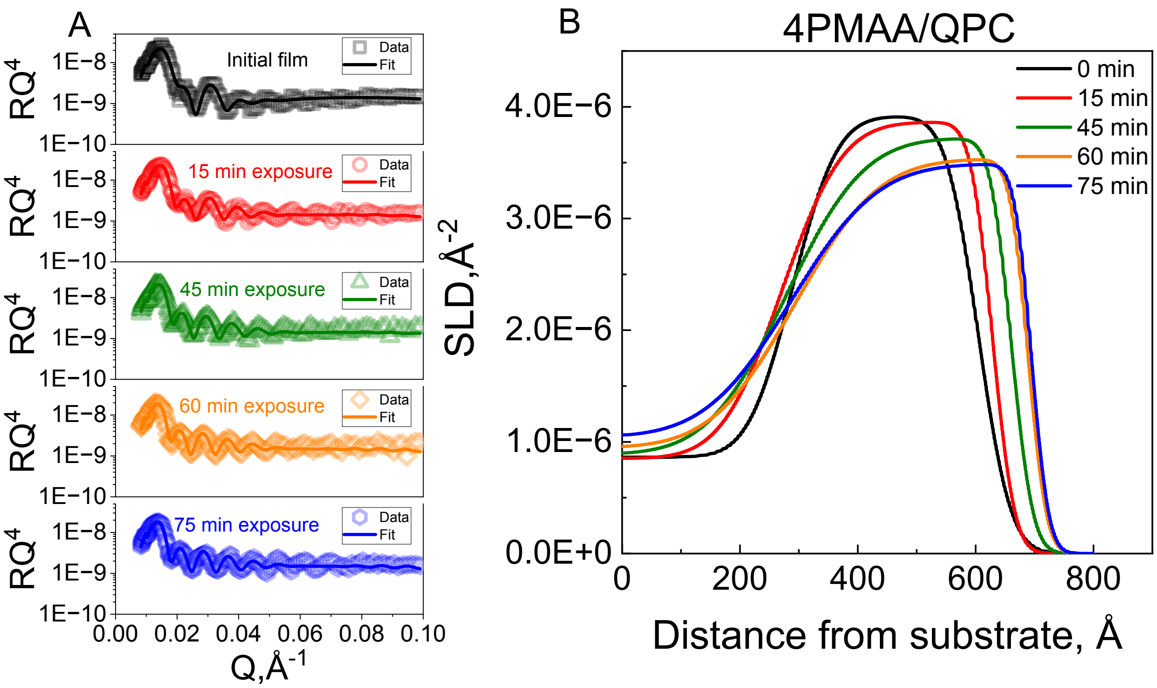

Fig. S9. (A) Neutron reflectivity data (plotted as RQ^4 to enhance small features) and (B) the corresponding neutron scattering length density profiles for 4PMAA/(*h*QPC/4PMAA)$_3$/(*d*QPC/4PMAA)$_3$ LbL film deposited at pH 6 before and after exposure to 0.25 M NaCl solutions.

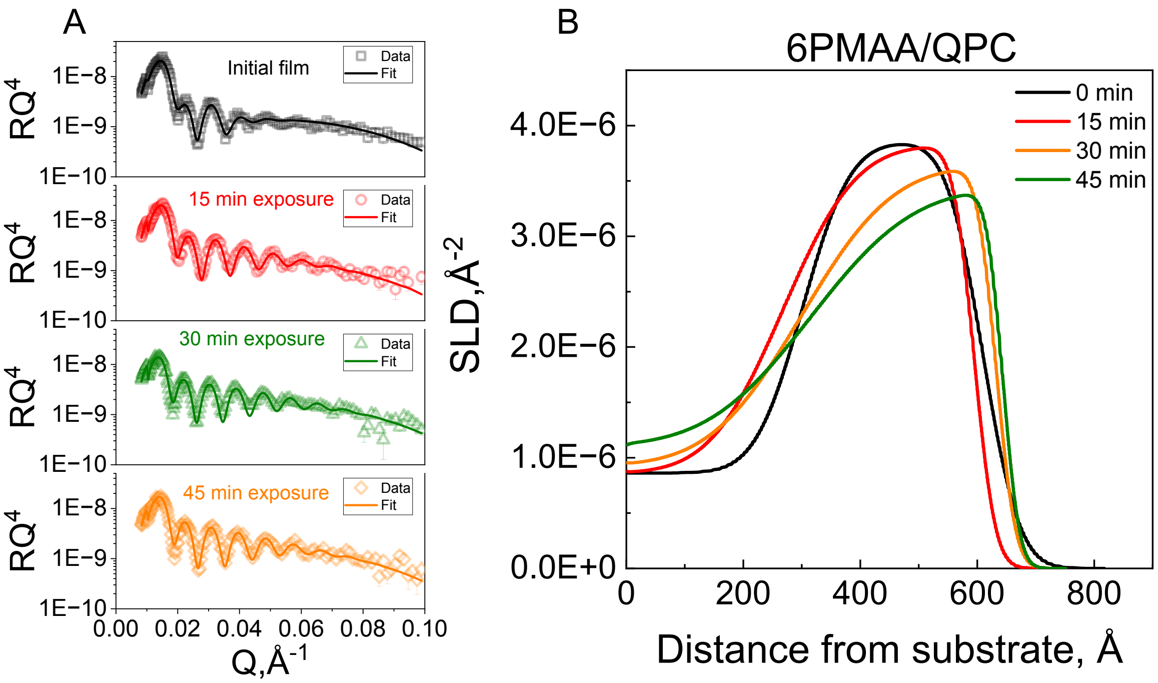

Fig. S10. (A) Neutron reflectivity data (plotted as RQ4 to enhance small features) and (B) the corresponding neutron scattering length density profiles for 6PMAA/(*h*QPC/6PMAA)$_3$/(*d*QPC/6PMAA)$_3$ LbL film deposited at pH 6 before and after exposure to 0.25 M NaCl solutions.

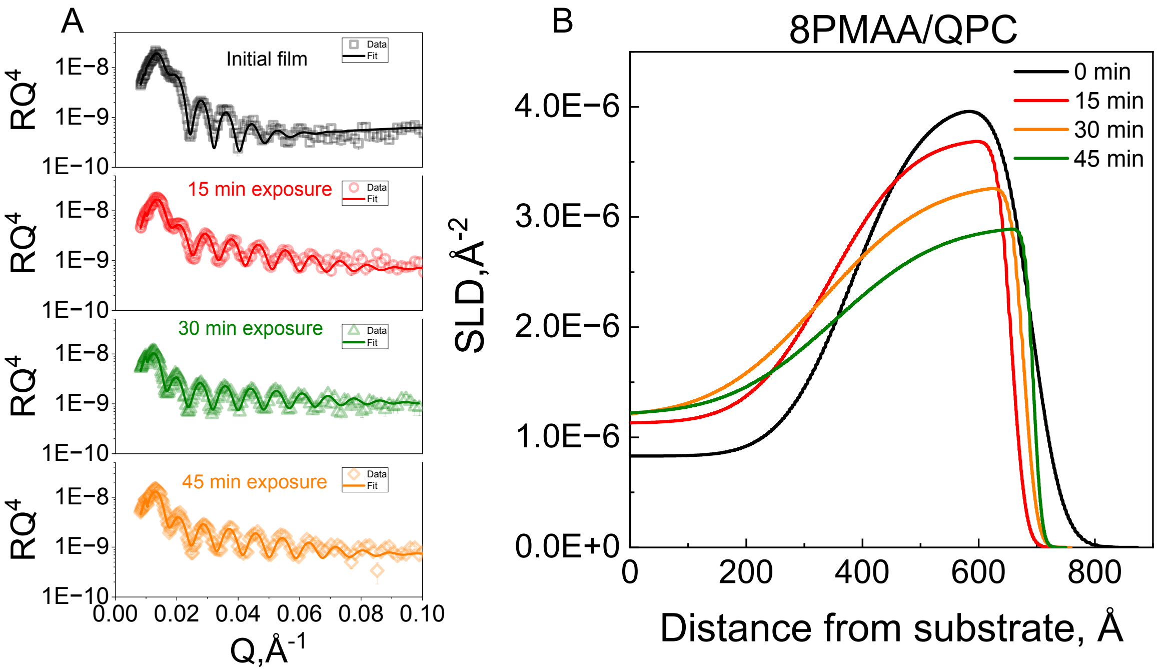

Fig. S11. (A) Neutron reflectivity data (plotted as RQ4 to enhance small features) and (B) the corresponding neutron scattering length density profiles for 8PMAA/(*h*QPC/8PMAA)$_3$/(*d*QPC/8PMAA)$_3$ LbL film deposited at pH 6 before and after exposure to 0.25 M NaCl solutions.

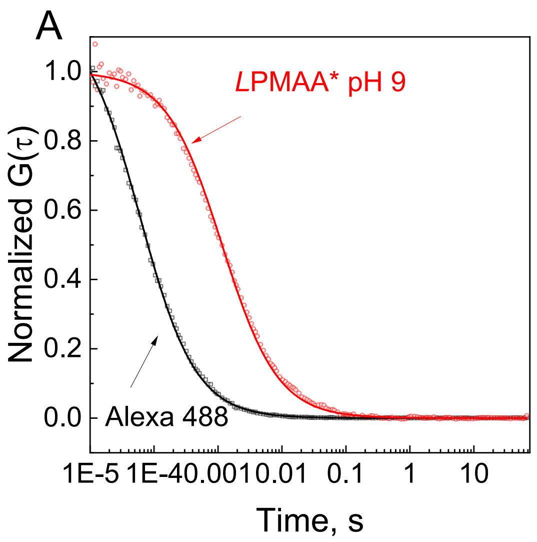

Fig. S12. The normalized autocorrelation functions for diffusion of LPMAA* (A) and 8PMAA (B) chains in 10^{-2} mg/ml solutions, along with diffusion of free Alexa-488 (4 nM concentration) in 0.01 M phosphate buffer at pH 9. The solid lines represent fits with the single component 3D diffusion model.

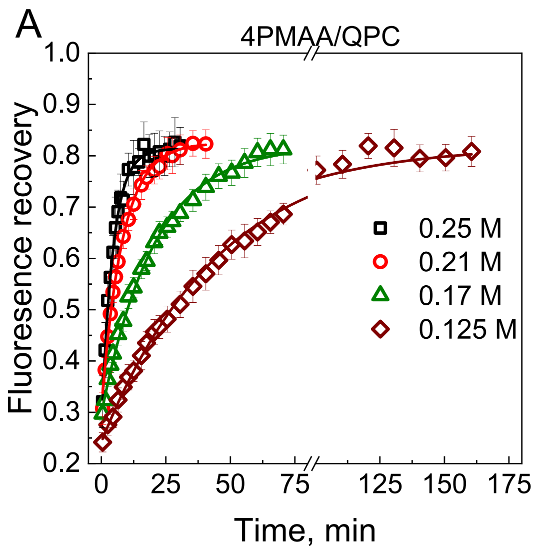

Fig. S13. Fluorescence recovery after photobleaching of $(QPC/4PMAA)_2/(QPC/4PMAA^*)_3/(QPC/4PMAA)_2$ (A) and $(QPC/6PMAA)_2/(QPC/6PMAA^*)_3/(QPC/6PMAA)_2$ (B) films deposited at pH 6 in 0.01 M phosphate buffer, bleached at 0.1 mW and monitored at 1μW when exposed to 0.125 M (diamonds), 0.17 M (up triangles), 0.21 M (circles) and 0.25 M (squares) NaCl solutions. The solid lines represent the exponential fit to the data.

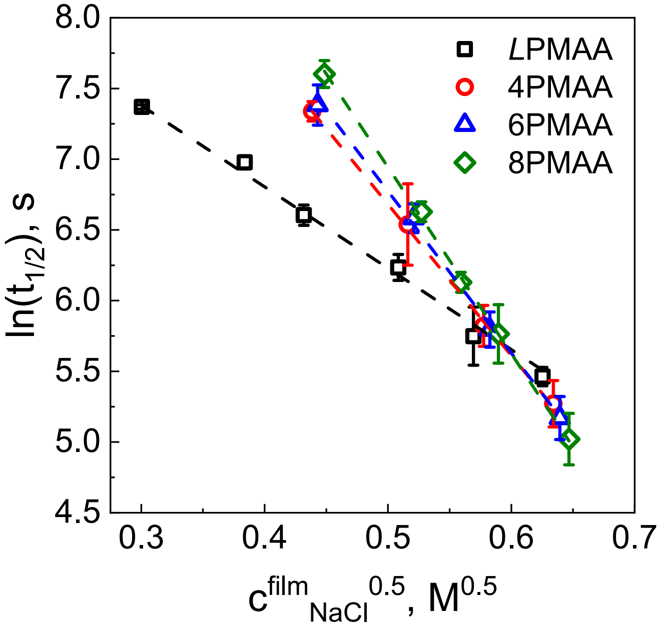

Fig. S14. Half-recovery time as a function of square root of apparent salt concentration (c_{NaCl}^{film}) in the swollen film for *L*PMAA*- (squares), 4PMAA* (circles), 6PMAA* (triangles), and 8PMAA*-containing (diamonds) films. The dashed lines represent linear fits to the data using the equation 4. The apparent salt concentration in the swollen film was calculated as follows: $c_{NaCl}^{film} = \frac{y * \frac{m_{film}}{M_{ion\,contact}} + V_{solv} * c_{NaCl}^{aq}}{V_{film}}$, where y is a doping level (fraction of externally compensated by salt charge in the film), $M_{ion\,contact}$ – molar weight of the ionic pair forming ionic contact (257 g/mol), m_{film} mass of the dry film in the swollen film (calculated from swelling data, Fig. 2), V_{solv} – volume of water in a swollen film, c_{NaCl}^{aq} – concentration of NaCl in aqueous solution, and V_{film} – total volume of the swollen film. Doping level was calculated as follows[4]: $y = \frac{w_{NaCl}^{aq}}{w_{film}} * \frac{M_{ion\,contact}}{M_{NaCl}}$, where w_{NaCl}^{aq} – weight percent of sodium chloride in solution, w_{film} – weight percent of dry film in the swollen film (calculated from swelling data, Fig. 2), M_{NaCl} – molar weight of NaCl.

Table S1. Diffusional exponent n obtained from the fitting of the kinetics of swelling using the equation $\frac{M_t}{M_\infty} = k_1 * t^n$ for (QPC/*L*PMAA)$_{20}$, (QPC/4PMAA)$_{20}$, (QPC/6PMAA)$_{20}$ and (QPC/8PMAA)$_{20}$ LbL films at different salt concentrations.

Salt concentration	*L*PMAA	4PMAA	6PMAA	8PMAA
0.05 M NaCl	1.6 ± 0.2	1.3 ± 0.2	1.1 ± 0.1	1.3 ± 0.1
0.1 M NaCl	1.2 ± 0.1	1.2 ± 0.1	1.2 ± 0.1	1.0 ± 0.1
0.15 M NaCl	1.1 ± 0.1	1.1 ± 0.1	1.2 ± 0.1	1.1 ± 0.1
0.2 M NaCl	1.2 ± 0.1	1.2 ± 0.1	1.3 ± 0.2	1.2 ± 0.1

Fitting Model for Neutron Reflectometry Data

We implemented the model previously used for analyzing the neutron data.[1, 2] In this model, we fitted the thickness and internal width of the hydrogenated and deuterated stacks (H- and D-stack respectively) as independent paraments, and scattering length density (SLD) of the stacks were parameterized by the fitting of film density, water content, ratio of polyanion to polycation and the intermixing between the stacks. Note that fitted internal roughness (**Table S3-S21**) are given as full width at half-maximum and are decreased by a factor 2.35 to get a Gaussian width that is used for the calculation of diffusion coefficients.

The SLD of a hydrogenated layer **(**$\Sigma_{\text{H - stack}}$) was calculated as follows:

$$\Sigma_{\text{H - stack}} = \rho_f \times \left\{ w_{H_2O} S_{H_2O} + (1 - w_{H_2O}) \times \left[f_{QPC} \times \left(w_{dQPC} \frac{M_{dQPC}}{M_{hQPC}} S_{dQPC} + \{1 - w_{dQPC}\} S_{hQPC} \right) + (1 - f_{QPC}) S_{PMAA} \right] \right\} \quad \text{(S1)},$$

The SLD of a deuterated layer **(**$\boldsymbol{\Sigma_{\text{D - stack}}}$) was calculated as follows:

$$\Sigma_{\text{D - stack}} = \rho_f \times \left\{ w_{H_2O} S_{H_2O} + (1 - w_{H_2O}) \times \left[f_{QPC} \times \left(w_{dQPC} S_{hQPC} + \{1 - w_{dQPC}\} S_{dQPC} \frac{M_{dQPC}}{M_{hQPC}} \right) + (1 - f_{QPC}) S_{PM} \right. \right. \quad \text{(S2)},$$

Where ρ_f is mass density of the film (g/cm^3), w_{H_2O} is the molar fraction of water in the film, and S_{H_2O}, S_{dQPC}, S_{hQPC}, and S_{PMAA} are normalized scattering length densities of water, *d*QPC, *h*QPC and linear or star PMAA respectively (**Table S2**) (Å^{-2} g^{-1} cm^3). f_{QPC} is the molar fraction of QPC in the polymer portion (PMAA/QPC) of the film. w_{dQPC} is the molar fraction of *d*QPC of the total QPC content of the H-stack, and M_{dQPC} and M_{hQPC} are molecular weights of the units of deuterated

and hydrogenated PDMAEMAs, respectively. The M_{dQPC}/M_{hQPC} ratio accounts for the increase in mass density with increasing fraction of deuterated material in the H-stack.

Table S2. Normalized scattering length densities for polymers and water used for fitting the neutron reflectivity data.

	PMAA	*h*QPC	*dQPC*	H_2O
S, Å^{-2} g^{-1} cm^{3}	1.1E-06	4.71E-07	6.36E-06	-5.60E-07

We also took into account the inhomogeneity of thickness of LbL films that causes a smearing of the reflectivity features at higher Q values[5] by using the following formula:

$$R_{inc} = (R_- + 2R_0 + R_+)/4 \tag{S3}$$

where R_0 is the specular reflectivity of the film using the nominal thicknesses of the LbL polymer layers and R_+ and R_- are the reflectivities of films in which the thicknesses are increased or decreased by δd, respectively.

Table S3. Model parameters for a *L*PMAA/(*h*QPC/*L*PMAA)$_4$/(*d*QPC/*L*PMAA)$_3$ film deposited from a 0.01 M phosphate buffer solution at pH 6.

**Layer**	***Nb*, Å^{-2}**	***d**, Å**	**σ_{int}*, Å**	**$\delta d/d$***
**(*d*QPC/*L*PMAA)$_3$**	3.91e-6	342.2±7	98.5±7	0.033±0.026
**(*h*QPC/*L*PMAA)$_4$**	8.65e-7	335.5±8	128±12	
**BPEI**	(9.8±2)e-7	27.1±6	25.7	N/A
**SiO_2**	(3.3±0.1)e-6	47.2±4	5	
**Si**	2.07e-6	100	20	
**Layer**	***f_{QPC}****	***w_{dQPC}****	***w_{H_2O}****	***ρ_f**, g/cm^3**
**(*d*QPC/*L*PMAA)$_3$**	0.44±0.02	0.00	0.03±0.03	1.09±0.04
**(*h*QPC/*L*PMAA)$_4$**				
**BPEI**	N/A	N/A		
**SiO_2**			N/A	N/A
**Si**				

*Fitted parameters

Table S4. Model parameters for a *L*PMAA/(*h*QPC/*L*PMAA)$_4$/(*d*QPC/*L*PMAA)$_3$ film after a 15-min exposure to 0.25 M NaCl solution at pH 6.

**Layer**	***Nb*, Å^{-2}**	***d**, Å**	**σ_{int}*, Å**	**$\delta d/d$***
**(*d*QPC/*L*PMAA)$_3$**	3.92e-6	349.2±16	90.7±10	0.083±0.037
**(*h*QPC/*L*PMAA)$_4$**	8.65e-7	330.5±8	158.3±19	
**BPEI**	(9.8±2)e-7	25.7±7	25.7	N/A
**SiO_2**	(3.3±0.1)e-6	47.2±4	5	
**Si**	2.07e-6	100	20	
**Layer**	***f_{QPC}****	***w_{dQPC}****	***w_{H_2O}****	***ρ_f*, g/cm^3***
**(*d*QPC/*L*PMAA)$_3$**	0.44±0.02	0.00	0.03±0.03	1.09±0.05
**(*h*QPC/*L*PMAA)$_4$**				
**BPEI**	N/A	N/A		
**SiO_2**			N/A	N/A
**Si**				

*Fitted parameters

Table S5. Model parameters for a *L*PMAA/(*h*QPC/*L*PMAA)$_4$/(*d*QPC/*L*PMAA)$_3$ film after a 45-min exposure to 0.25 M NaCl solution at pH 6.

**Layer**	***Nb*, Å^{-2}**	***d**, Å**	**σ_{int}*, Å**	**$\delta d/d$***
**(*d*QPC/*L*PMAA)$_3$**	3.66e-6	377.8±22	54.8±11	0.061±0.018
**(*h*QPC/*L*PMAA)$_4$**	7.96e-7	343.4±10	207.6±27	
**BPEI**	(9.8±2)e-7	25.7±7	25.7	N/A
**SiO_2**	(3.3±0.1)e-6	47.2±4	5	
**Si**	2.07e-6	100	20	
**Layer**	***f_{QPC}****	***w_{dQPC}****	***w_{H_2O}****	***ρ_f*, g/cm^3***
**(*d*QPC/*L*PMAA)$_3$**	0.44±0.02	0.00	0.05±0.02	1.05±0.04
**(*h*QPC/*L*PMAA)$_4$**				
**BPEI**	N/A	N/A		
**SiO_2**			N/A	N/A
**Si**				

*Fitted parameters

Table S6. Model parameters for a *L*PMAA/(*h*QPC/*L*PMAA)$_4$/(*d*QPC/*L*PMAA)$_3$ film after a 60-min exposure to 0.25 M NaCl solution at pH 6.

**Layer**	***Nb*, Å^{-2}**	***d**, Å**	**σ_{int}*, Å**	**$\delta d/d$***
**(*d*QPC/*L*PMAA)$_3$**	3.53e-6	397.7±23	67.3±13	0.05±0.037
**(*h*QPC/*L*PMAA)$_4$**	7.61e-7	353.7±10	219.3±30	
**BPEI**	(9.8±2)e-7	25.7±7	25.7	N/A
**SiO_2**	(3.3±0.1)e-6	47.2±4	5	
**Si**	2.07e-6	100	20	
**Layer**	***f_{QPC}****	***w_{dQPC}****	***w_{H_2O}****	***ρ_f*, g/cm^3***
**(*d*QPC/*L*PMAA)$_3$**	0.44±0.02	0.00	0.07±0.03	1.05±0.04
**(*h*QPC/*L*PMAA)$_4$**				
**BPEI**	N/A	N/A		
**SiO_2**			N/A	N/A
**Si**				

*Fitted parameters

Table S7. Model parameters for a *L*PMAA/(*h*QPC/*L*PMAA)$_4$/(*d*QPC/*L*PMAA)$_3$ film after a 75-min exposure to 0.25 M NaCl solution at pH 6.

**Layer**	***Nb*, Å^{-2}**	***d**, Å**	**σ_{int}*, Å**	**$\delta d/d$***
**(*d*QPC/*L*PMAA)$_3$**	3.59e-6	392.5±33	73.3±11	0.085±0.020
**(*h*QPC/*L*PMAA)$_4$**	8.01e-7	335.8±12	236.2±32	
**BPEI**	(9.8±2)e-7	25.7±7	25.7	N/A
**SiO_2**	(3.3±0.1)e-6	47.2±4	5	
**Si**	2.07e-6	100	20	
**Layer**	***f_{QPC}****	***w_{dQPC}****	***w_{H_2O}****	***ρ_f*, g/cm^3***
**(*d*QPC/*L*PMAA)$_3$**	0.44±0.02	0.01±0.01	0.07±0.03	1.06±0.03
**(*h*QPC/*L*PMAA)$_4$**				
**BPEI**	N/A	N/A		
**SiO_2**			N/A	N/A
**Si**				

*Fitted parameters

Table S8. Model parameters for a 4PMAA/(*h*QPC/4PMAA)$_3$/(*d*QPC/4PMAA)$_3$ film deposited from a 0.01 M phosphate buffer solution at pH 6.

Layer	*Nb*, Å^{-2}	*d**, Å	σ_{int}*, Å	*δd/d**
**(*d*QPC/4PMAA)$_3$**	3.92e-6	315.1±8	105.7±10	0.077±0.037
**(*h*QPC/4PMAA)$_3$**	8.62e-7	323.1±10	139.6±13	
**BPEI**	(11±2)e-7	24±10	24	N/A
**SiO_2**	(3.3±0.1)e-6	32.3±3	5	
**Si**	2.07e-6	100	20	
**Layer**	***f_{QPC}****	***w_{dQPC}****	***w_{H_2O}****	***ρ_f*, g/cm^3***
**(*d*QPC/4PMAA)$_3$**	0.44±0.02	0.00	0.03±0.03	1.1±0.05
**(*h*QPC/4PMAA)$_3$**				
**BPEI**	N/A	N/A		
**SiO_2**			N/A	N/A
**Si**				

*Fitted parameters

Table S9. Model parameters for a 4PMAA/(*h*QPC/4PMAA)$_3$/(*d*QPC/4PMAA)$_3$ film after a 15-min exposure to 0.25 M NaCl solution at pH 6.

Layer	*Nb*, Å^{-2}	*d**, Å	σ_{int}*, Å	*δd/d**
**(*d*QPC/4PMAA)$_3$**	3.86e-6	357.3±30	69±8	0.077±0.037
**(*h*QPC/4PMAA)$_3$**	8.51e-7	295.4±12	190.6±20	
**BPEI**	(11±2)e-7	24±10	24	N/A
**SiO_2**	(3.3±0.1)e-6	32.3±3	5	
**Si**	2.07e-6	100	20	
**Layer**	***f_{QPC}****	***w_{dQPC}****	***w_{H_2O}****	***ρ_f*, g/cm^3***
**(*d*QPC/4PMAA)$_3$**	0.44±0.02	0.00	0.03±0.03	1.08±0.04
**(*h*QPC/4PMAA)$_3$**				
**BPEI**	N/A	N/A		
**SiO_2**			N/A	N/A
**Si**				

*Fitted parameters

Table S10. Model parameters for a 4PMAA/(*h*QPC/4PMAA)$_3$/(*d*QPC/4PMAA)$_3$ film after a 45-min exposure to 0.25 M NaCl solution at pH 6.

**Layer**	***Nb*, Å^{-2}**	***d**, Å**	**σ_{int}*, Å**	**$\delta d/d$***
**(*d*QPC/4PMAA)$_3$**	3.72e-6	383±32	67.5±7	0.071±0.022
**(*h*QPC/4PMAA)$_3$**	8.88e-7	302.6±10	246±25	
**BPEI**	(11±2)e-7	24±10	24	N/A
**SiO_2**	(3.3±0.1)e-6	32.3±3	5	
**Si**	2.07e-6	100	20	
**Layer**	**f_{QPC}***	**w_{dQPC}***	**w_{H_2O}***	**ρ_f*, g/cm^3**
**(*d*QPC/4PMAA)$_3$**	0.44±0.02	0.02±0.02	0.03±0.03	1.06±0.03
**(*h*QPC/4PMAA)$_3$**				
**BPEI**	N/A	N/A		
**SiO_2**			N/A	N/A
**Si**				

*Fitted parameters

Table S11. Model parameters for a 4PMAA/(*h*QPC/4PMAA)$_3$/(*d*QPC/4PMAA)$_3$ film after a 60-min exposure to 0.25 M NaCl solution at pH 6.

**Layer**	***Nb*, Å^{-2}**	***d**, Å**	**σ_{int}*, Å**	**$\delta d/d$***
**(*d*QPC/4PMAA)$_3$**	3.53e-6	395.1±33	58.1±9	0.071±0.022
**(*h*QPC/4PMAA)$_3$**	9.47e-7	320±10	263.8±28	
**BPEI**	(11±2)e-7	24±10	24	N/A
**SiO_2**	(3.3±0.1)e-6	32.3±3	5	
**Si**	2.07e-6	100	20	
**Layer**	**f_{QPC}***	**w_{dQPC}***	**w_{H_2O}***	**ρ_f*, g/cm^3**
**(*d*QPC/4PMAA)$_3$**	0.44±0.02	0.05±0.03	0.04±0.03	1.04±0.03
**(*h*QPC/4PMAA)$_3$**				
**BPEI**	N/A	N/A		
**SiO_2**			N/A	N/A
**Si**				

*Fitted parameters

Table S12. Model parameters for a 4PMAA/(*h*QPC/4PMAA)$_3$/(*d*QPC/4PMAA)$_3$ film after a 75-min exposure to 0.25 M NaCl solution at pH 6.

Layer	*Nb*, Å^{-2}	*d**, Å	σ_{int}*, Å	*δd/d**
**(*d*QPC/4PMAA)$_3$**	3.49e-6	407.6±38	54.6±9	0.069±0.019
**(*h*QPC/4PMAA)$_3$**	1.04e-6	302.1±11	272.4±30	
**BPEI**	(11±2)e-7	24±10	24	N/A
**SiO_2**	(3.3±0.1)e-6	32.3±3	5	
**Si**	2.07e-6	100	20	
**Layer**	f_{QPC}*	w_{dQPC}*	w_{H_2O}*	ρ_f*, g/cm^3
**(*d*QPC/4PMAA)$_3$**	0.44±0.02	0.08±0.03	0.05±0.03	1.04±0.03
**(*h*QPC/4PMAA)$_3$**				
**BPEI**	N/A	N/A		
**SiO_2**			N/A	N/A
**Si**				

*Fitted parameters

Table S13. Model parameters for a 6PMAA/(*h*QPC/6PMAA)$_3$/(*d*QPC/6PMAA)$_3$ film deposited from a 0.01 M phosphate buffer solution at pH 6.

Layer	*Nb*, Å^{-2}	*d**, Å	σ_{int}*, Å	*δd/d**
**(*d*QPC/6PMAA)$_3$**	3.85e-6	308.5±9	116±7	0.062±0.031
**(*h*QPC/6PMAA)$_3$**	8.62e-7	327.4±10	150.5±15	
**BPEI**	(9.8±2)e-7	25.3±8	25.3	N/A
**SiO_2**	(3.2±0.1)e-6	48±7	25	
**Si**	2.07e-6	100	20	
**Layer**	f_{QPC}*	w_{dQPC}*	w_{H_2O}*	ρ_f*, g/cm^3
**(*d*QPC/6PMAA)$_3$**	0.43±0.02	0.00	0.03±0.03	1.07±0.04
**(*h*QPC/6PMAA)$_3$**				
**BPEI**	N/A	N/A		
**SiO_2**			N/A	N/A
**Si**				

*Fitted parameters

Table S14. Model parameters for a 6PMAA/(*h*QPC/6PMAA)$_3$/(*d*QPC/6PMAA)$_3$ film after a 15-min exposure to 0.25 M NaCl solution at pH 6.

Layer	*Nb*, Å^{-2}	*d**, Å	σ_{int}*, Å	*δd/d**
**(*d*QPC/6PMAA)$_3$**	3.82e-6	326.2±31	67.3±11	0.057±0.031
**(*h*QPC/6PMAA)$_3$**	8.6e-7	285.3±7	231.3±25	
**BPEI**	(9.8±2)e-7	25.3±8	25.3	N/A
**SiO_2**	(3.2±0.1)e-6	48±7	25	
**Si**	2.07e-6	100	20	
**Layer**	*f_{QPC}**	*w_{dQPC}**	*w_{H_2O}**	*ρ_f*, g/cm^3*
**(*d*QPC/6PMAA)$_3$**	0.43±0.02	0.001±0.01	0.03±0.03	1.07±0.04
**(*h*QPC/6PMAA)$_3$**				
**BPEI**	N/A	N/A		
**SiO_2**			N/A	N/A
**Si**				

*Fitted parameters

Table S15. Model parameters for a 6PMAA/(*h*QPC/6PMAA)$_3$/(*d*QPC/6PMAA)$_3$ film after a 30-min exposure to 0.25 M NaCl solution at pH 6.

Layer	*Nb*, Å^{-2}	*d**, Å	σ_{int}*, Å	*δd/d**
**(*d*QPC/6PMAA)$_3$**	3.64e-6	332.4±33	61±13	0.051±0.019
**(*h*QPC/6PMAA)$_3$**	9.27e-7	311.6±8	291.1±28	
**BPEI**	(9.8±2)e-7	25.3±8	25.3	N/A
**SiO_2**	(3.2±0.1)e-6	48±7	25	
**Si**	2.07e-6	100	20	
**Layer**	*f_{QPC}**	*w_{dQPC}**	*w_{H_2O}**	*ρ_f*, g/cm^3*
**(*d*QPC/6PMAA)$_3$**	0.43±0.02	0.03±0.02	0.04±0.03	1.04±0.03
**(*h*QPC/6PMAA)$_3$**				
**BPEI**	N/A	N/A		
**SiO_2**			N/A	N/A
**Si**				

*Fitted parameters

Table S16. Model parameters for a 6PMAA/(*h*QPC/6PMAA)$_3$/(*d*QPC/6PMAA)$_3$ film after a 45-min exposure to 0.25 M NaCl solution at pH 6.

Layer	*Nb*, Å^{-2}	*d**, Å	σ_{int}*, Å	*δd/d**
**(*d*QPC/6PMAA)$_3$**	3.45e-6	328±35	52.5±8	0.051±0.019
**(*h*QPC/6PMAA)$_3$**	1.1e-6	328.2±7	328±35	
**BPEI**	(9.8±2)e-7	25.3±8	25.3	N/A
**SiO_2**	(3.2±0.1)e-6	48±7	25	
**Si**	2.07e-6	100	20	
**Layer**	***f*$_{QPC}$***	***w*$_{dQPC}$***	***w*$_{H_2O}$***	***ρ*$_f$*, g/cm^3**
**(*d*QPC/6PMAA)$_3$**	0.43±0.02	0.09±0.03	0.04±0.03	1.04±0.03
**(*h*QPC/6PMAA)$_3$**				
**BPEI**	N/A	N/A		
**SiO_2**			N/A	N/A
**Si**				

*Fitted parameters

Table S17. Model parameters for a 8PMAA/(*h*QPC/8PMAA)$_3$/(*d*QPC/8PMAA)$_3$ film deposited from a 0.01 M phosphate buffer solution at pH 6.

Layer	*Nb*, Å^{-2}	*d**, Å	σ_{int}*, Å	*δd/d**
**(*d*QPC/8PMAA)$_3$**	4.02e-6	308±20	99.5±6	0.04±0.027
**(*h*QPC/8PMAA)$_3$**	8.25e-7	407.6±10	226.1±20	
**BPEI**	(8.8±2)e-7	20.7±7	20.7	N/A
**SiO_2**	(3.2±0.1)e-6	12.6±3	10	
**Si**	2.07e-6	100	20	
**Layer**	***f*$_{QPC}$***	***w*$_{dQPC}$***	***w*$_{H_2O}$***	***ρ*$_f$*, g/cm^3**
**(*d*QPC/8PMAA)$_3$**	0.46±0.03	0.00	0.05±0.03	1.12±0.05
**(*h*QPC/8PMAA)$_3$**				
**BPEI**	N/A	N/A		
**SiO_2**			N/A	N/A
**Si**				

*Fitted parameters

Table S18. Model parameters for a 8PMAA/(*h*QPC/8PMAA)$_3$/(*d*QPC/8PMAA)$_3$ film after a 15-min exposure to 0.25 M NaCl solution at pH 6.

Layer	*Nb*, Å^{-2}	*d**, Å	σ_{int}*, Å	*δd/d**
**(*d*QPC/8PMAA)$_3$**	3.72e-6	312.5±30	45±7	0.04±0.026
**(*h*QPC/8PMAA)$_3$**	1.13e-6	365.7±9	258±30	
**BPEI**	(8.8±2)e-7	20.7±7	20.7	N/A
**SiO_2**	(3.2±0.1)e-6	12.6±3	10	
**Si**	2.07e-6	100	20	

Layer	f_{QPC}*	w_{dQPC}*	w_{H_2O}*	ρ_f*, g/cm^3
**(*d*QPC/8PMAA)$_3$**	0.46±0.03	0.1±0.03	0.05±0.03	1.11±0.04
**(*h*QPC/8PMAA)$_3$**				
**BPEI**	N/A	N/A		
**SiO_2**			N/A	N/A
**Si**				

*Fitted parameters

Table S19. Model parameters for a 8PMAA/(*h*QPC/8PMAA)$_3$/(*d*QPC/8PMAA)$_3$ film after a 30-min exposure to 0.25 M NaCl solution at pH 6.

Layer	*Nb*, Å^{-2}	*d**, Å	σ_{int}*, Å	*δd/d**
**(*d*QPC/8PMAA)$_3$**	3.31e-6	347.4±34	41±8	0.04±0.026
**(*h*QPC/8PMAA)$_3$**	1.19e-6	343.4±28	343.4±28	
**BPEI**	(8.8±2)e-7	20.7±7	20.7	N/A
**SiO_2**	(3.2±0.1)e-6	12.6±3	10	
**Si**	2.07e-6	100	20	

Layer	f_{QPC}*	w_{dQPC}*	w_{H_2O}*	ρ_f*, g/cm^3
**(*d*QPC/8PMAA)$_3$**	0.46±0.03	0.14±0.03	0.05±0.03	1.04±0.04
**(*h*QPC/8PMAA)$_3$**				
**BPEI**	N/A	N/A		
**SiO_2**			N/A	N/A
**Si**				

*Fitted parameters

Table S20. Model parameters for a 8PMAA/(*h*QPC/8PMAA)$_3$/(*d*QPC/8PMAA)$_3$ film after a 30-min exposure to 0.25 M NaCl solution at pH 6.

Layer	*Nb*, Å^{-2}	*d**, Å	σ_{int}*, Å	*δd/d**
**(*d*QPC/8PMAA)$_3$**	2.92e-6	341.2±37	26.5±5	0.04±0.026
**(*h*QPC/8PMAA)$_3$**	1.21e-6	375.4±24	341.2±37	
**BPEI**	(8.8±2)e-7	20.7±7	20.7	N/A
**SiO_2**	(3.2±0.1)e-6	12.6±3	10	
**Si**	2.07e-6	100	20	
**Layer**	***f*$_{QPC}$***	***w*$_{dQPC}$***	***w*$_{H_2O}$***	***ρ*$_f$*, g/cm^3**
**(*d*QPC/8PMAA)$_3$**	0.46±0.03	0.19±0.03	0.09±0.03	1.01±0.03
**(*h*QPC/8PMAA)$_3$**				
**BPEI**	N/A	N/A		
**SiO_2**			N/A	N/A
**Si**				

Table S21. Internal roughness between *H*- and *D*-stack and the molar fraction of *d*QPC of the total QPC content of the *H*-stack (w_{dQPC}) for PMAA/QPC films at different time of annealing.

	*L*PMAA/QPC		4PMAA/QPC		6PMAA/QPC		8PMAA/QPC	
Annealing time, min	σ_{int}, Å	w_{dQPC}	σ_{int}, Å	w_{dQPC}	σ_{int}, Å	w_{dQPC}	σ_{int}, Å	w_{dQPC}
Initial	128±12	0	140±13	0	116±7	0	226±20	0
15 min	158±19	0	191±20	0	231±25	0	258±30	0.1±0.03
30 min	N/A	N/A	N/A	N/A	291±28	0.03±0.02	343±28	0.14±0.03
45 min	208±27	0	246±25	0.02±0.02	328±35	0.09±0.03	341±37	0.19±0.03
60 min	219±30	0	264±28	0.05±0.03	N/A	N/A	N/A	N/A
75 min	236±32	0.01±0.01	272±30	0.08±0.03	N/A	N/A	N/A	N/A

Table S22. Half-time of fluorescent recovery and diffusion coefficients obtained from FRAP experiments for linear and star PMAA/QPC films at different salt concentrations.

	*L*PMAA/QPC		4PMAA/QPC		6PMAA/QPC		8PMAA/QPC	
c_{NaCl}, M	$t_{1/2}$, min	D, 10^{-13} cm^2/s	$t_{1/2}$, min	D, 10^{-13} cm^2/s	$t_{1/2}$, min	D, 10^{-13} cm^2/s	$t_{1/2}$, min	D, 10^{-13} cm^2/s
0.0625	26.5±0.4	1.1±0.1	N/A	N/A	N/A	N/A	N/A	N/A
0.1	17.9±0.8	1.6±0.1	N/A	N/A	N/A	N/A	N/A	N/A
0.125	12.2±0.8	2.3±-0.2	25.7±1.8	1.2±0.08	26.8±1.4	1.1±0.1	33.5±3	0.9±0.1
0.17	8.5±0.8	3.4±0.3	11.9±3.2	2.6±0.8	12.1±1.2	2.5±0.3	12.6±0.9	2.3±0.2
0.19	N/A	N/A	N/A	N/A	N/A	N/A	7.7±0.5	3.8±0.3
0.21	5.5±1	5.6±1.2	5.7±0.8	5.2±0.8	5.5±0.7	5.3±0.7	5.4±1.1	5.5±0.1
0.25	3.9±0.3	7.3±0.5	3.3±0.5	8.9±1.6	2.9±0.4	9.9±1.6	2.5±0.5	11.5±2

References:

1. Aliakseyeu, A.; Albright, V.; Yarbrough, D.; Hernandez, S.; Zhou, Q.; Ankner, J. F.; Sukhishvili, S. A., Selective hydrogen bonding controls temperature response of layer-by-layer upper critical solution temperature micellar assemblies. *Soft Matter* **2021,** *17* (8), 2181-2190.
2. Aliakseyeu, A.; Ankner, J. F.; Sukhishvili, S. A., Impact of Star Polyacid Branching on Polymer Diffusion within Multilayer Films. *Macromolecules* **2022,** *55* (18), 8150-8161.
3. Tanchak, O. M.; Barrett, C. J., Swelling Dynamics of Multilayer Films of Weak Polyelectrolytes. *Chemistry of Materials* **2004,** *16* (14), 2734-2739.
4. Yang, M.; Shi, J.; Schlenoff, J. B., Control of Dynamics in Polyelectrolyte Complexes by Temperature and Salt. *Macromolecules* **2019,** *52* (5), 1930-1941.
5. Hlushko, R.; Ankner, J. F.; Sukhishvili, S., Dynamics and Self-Healing of Layer-by-Layer Hydrogen-Bonded Films of Linear Synthetic Polyphenols. *Macromolecules* **2021,** *54* (16), 7469-7479.
